# A Conventional Framework for Reflection Removal Using Single Images

**DOI:** 10.3390/jimaging12090439

**Published:** 2026-09-12

**Authors:** Mehwish Arif, Khawar Khurshid, Muhammad Shahzeb Khan Gul

**Affiliations:** 1 School of Electrical Engineering and Computer Sciences, National University of Sciences and Technology, Islamabad 44000, Pakistan; marif.msee16seecs@seecs.edu.pk; 2 Department of Computer Science, Namal University, Mianwali 42250, Pakistan; khawar.khurshid@namal.edu.pk; 3 Fraunhofer Institute for Integrated Circuits, 91058 Erlangen, Germany

**Keywords:** two-stage thresholding, reflection removal, perceptual image quality, structure layer, texture layer, soft matting

## Abstract

Reflections caused by poor lighting conditions or the presence of transparent media such as glass often degrade image quality by introducing unwanted visual artifacts and obscuring important scene information. These reflections not only reduce the perceptual quality of images but also adversely affect the performance of downstream computer vision applications. Reflection removal is therefore a critical pre-processing step for enhancing image quality and improving the reliability of vision-based systems. In this paper, we propose a pipeline framework that employs individual components from established prior works on reflection removal. The proposed method uses a hierarchical thresholding consisting of two stages to extract a reflection-free structure layer. DCT and soft matting suppress reflection components in the texture layer while preserving important image details. The performance of the proposed approach was evaluated on a benchmark dataset developed by ROSE Lab at Nanyang Technological University, Singapore. Experimental results, assessed using multiple quantitative and perceptual quality metrics, demonstrate that the proposed method effectively suppresses reflections, produces images with fewer unnatural artifacts, and achieves improved perceptual quality relative to the baseline methods evaluated in the study, based on no-reference metrics’ results. The competitive visual quality and natural appearance of the restored images suggest the proposed framework may be a promising candidate for pre-processing in computer vision applications.

## 1. Introduction

Reflections in images occur whenever an image is captured in poor lighting conditions or through a transparent medium like windows or other glass surfaces. The presence of reflections significantly degrades image quality. It not only causes poor visibility of the background scene but also makes the usability of images in computer vision tasks more difficult. In the real world, reflections and shadows are inevitable. Therefore, there is a need to remove these reflections and preserve background information as much as possible. Mathematically, such images can be modeled as a linear model [1], expressed below:

(1) I=B+R,

where *I* is the input image, *B* is the background layer, and *R* is the reflection layer.

Equation (1) shows the ill-posed nature of the problem. The number of unknown variables is twice the number of equations. With no additional knowledge, this expression gives an infinite number of solutions. To make it more tractable, additional information is required in the form of priors or constraints.

Existing methods have proposed various techniques to address reflection removal using single images. Professional photographers use crossed polarizers to solve this problem. Placing one polarizer in front of the light source while rotating the second one in front of the lens largely minimizes reflection. Another way is to capture images at two different angles of the polarizing lens and then take the linear combination of both [1]. Another solution is to use multiple images, in which the regions of reflection and transmission are exploited under different motion states. No matter how appealing or effective these methods are, their applicability at the consumer end is not economical.

Single-image reflection removal techniques provide a resource-constrained solution while presenting the challenge of working with minimal available information. Their suitability for consumers is the most researched problem. The most common observation, which also forms the base of our proposed method, is that the reflection layer contains some degree of blur or smoothness that separates it from the background layer. The prevalent approach uses this assumption to separate reflections from single images.

In this paper, we employ a two-stage hierarchical thresholding algorithm to remove as many reflections as possible without affecting background details. A TV regularization approach (which preserves sharp edges while smoothing noise) decomposes the image into texture and structure layers [2]. Reflections in the texture layer are then suppressed through a DCT-based detection scheme that identifies genuine high-frequency scene detail [3], followed by a soft-matting refinement step [4] guided by the structure layer, which estimates opacity values for precise reflection suppression. We hypothesize that our proposed method allows us to preserve maximum background details while removing as many reflections as possible.

**Figure 1:**
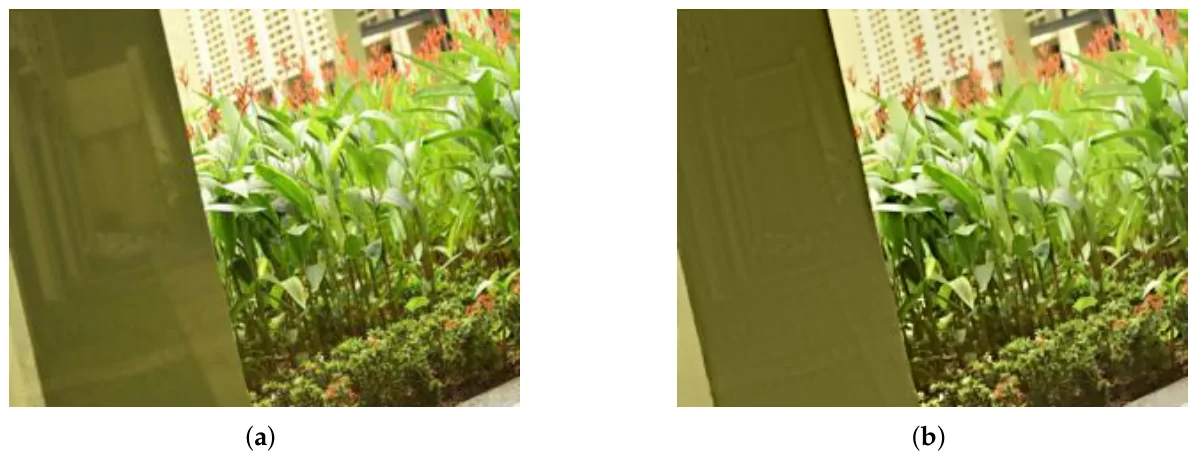
(**a**) A wild scene image, taken from the SIR2+ outdoor dataset [5] containing reflection artifacts. (**b**) Reflection removal using the proposed algorithm.

The results demonstrate that the proposed method is competitive with traditional single-image reflection removal approaches across quantitative metrics, including PSNR, SSIM, and perceptual quality measures, while achieving a clear advantage in perceptual quality. When compared to the deep learning baselines evaluated in this study, our approach achieves competitive pixel-level accuracy and structural similarity, while maintaining stronger perceptual quality with significantly better BRISQUE and PIQE scores, although this does not extend to the NIQE score. These results indicate that the proposed algorithm effectively suppresses reflections, produces images with fewer unnatural artifacts, and achieves favorable perceptual fidelity on a subset of no-reference metrics, avoiding GPU acceleration, large training datasets, and specialized hardware that deep learning counterparts require, allowing for deployment on general-purpose hardware. Figure 1 shows the images where reflections are removed using the proposed algorithm.

### 1.1. Related Work

Whenever an image is taken, special arrangements are necessary; otherwise, the image will be distorted by reflections caused by different lighting conditions or other transparent media in the vicinity. However, with the phenomenal increase in smartphone usage for taking images, arranging a special setup for every image is impractical. Therefore, we need to employ various pre-processing techniques. Pre-processing images for reflection removal can significantly enhance image quality with minimal to no loss of details.

Over the decades, many methods have been proposed to remove reflections from images. The broad categorization of reflection removal problems depends on the number of inputs (e.g., multiple images, single images). This categorization further branches into classification based on various constraints (e.g., motion cues, sparsity priors) [6,7], special capture setups (e.g., polarizers, lighting conditions, flash, focus) [8,9,10,11,12], exploitation of physical properties of both reflection and background layers (e.g., relative smoothness, ghosting effect) [13,14], and deep learning algorithms, where huge datasets are used for training.

#### 1.1.1. Multiple Images

Many algorithms rely on multiple images for reflection removal. Various constraints are exploited to achieve the best output quality. A vast majority of methods utilize motion cues between reflection and transmission layers of a scene with multi-view captures [15]. For example, refs. [6,16,17,18,19] estimate the motion field for each layer using SIFT flow for correct labeling of reflection and background layers. The difference in motion patterns between layers ranges from parametric static ones to generalized dense motion fields. Taking multiple images from various polarization angles allows us to estimate the background layer from the reflection layer [20,21]. A diverse range of other approaches exploit additional physical cues such as polarization and flash illumination to improve layer separation [10,11]. Wang et al. [12] propose a dual-stream attention-guided network that more effectively exploits polarization cues through adversarial training. Bian et al. [22] further explore a recurrent polarization-to-polarization network for improved temporal consistency across polarization captures. Statistical approaches, such as those in [23,24], treat reflection removal as a blind source-separation problem and estimate the mixing coefficients using independent component analysis (ICA).

#### 1.1.2. Single Images

Single-image reflection removal approaches pose a more difficult challenge, as no inter-image information is available. The ill-posed problem is made tractable by investigating several aspects of images and the background and reflection layers. The most widespread practice is to explore statistical properties of natural images. The authors of [1] stated that when derivative filters are applied to natural images, the output tends to be sparse. For instance, refs. [7,13,25,26,27] separated reflection and background layers by exploiting smoothness, gradient sparsity, and image statistical priors. Other approaches have modeled image statistics using mixture distributions [1,5]. Ghosting effect [16,28], relative smoothness [13,25], depth-of-field [29,30,31,32], and more recent reflection-free cue-based strategies [33] have also been exploited for separation of background and reflection layers.

Recent benchmark studies have further analyzed the performance of traditional and learning-based approaches under diverse reflection conditions [5]. Fan et al. [34] pioneered deep learning for reflection removal by proposing a deep convolutional neural network trained to predict edge information for separating both layers. Subsequent approaches [35,36,37] improved reflection removal performance using multi-scale networks, perceptual losses, adversarial training, and auxiliary information, while more recent methods have continued to advance the field through diffusion-transformer-based generalization to unseen reflection patterns [38], reversible decoupling architectures for improved structural reconstruction [39], and lightweight network designs that substantially reduce model complexity [40].

Despite noteworthy progress, several disagreements persist in the reflection removal literature. First, regarding image priors: some methods assume the reflection layer is inherently smoother or more blurred than the background [13,26], while others rely on sparsity priors, arguing that reflections appear primarily in high-frequency regions [1,7]. Second, a growing divide exists between deep learning and traditional optimization-based approaches. Deep learning methods [36,37,38,39] have achieved impressive results, but they often introduce unnatural artifacts and require substantial computational resources, large training datasets, and high-end hardware, making them impractical for deployment on resource-constrained platforms such as smartphones. Traditional methods, in contrast, are lightweight and device-agnostic, yet they have been criticized for underperforming in complex real-world scenes. Third, the community disagrees on evaluation standards: pixel-wise metrics like PSNR and SSIM favor methods that closely reconstruct synthetic ground truths, whereas no-reference perceptual metrics (BRISQUE, NIQE, PIQE) may better reflect human perception and artifact suppression in real-world images. This paper addresses these open questions by proposing a traditional, optimization-based method that demonstrates the following:

- Adopts the smoothness prior for reflections;
- Requires no GPU acceleration, large-scale training data, or specialized hardware, allowing for deployment on general-purpose consumer devices;
- Prioritizes perceptual quality over pixel-wise reconstruction accuracy.

## 2. Materials and Methods

**Figure 2:**
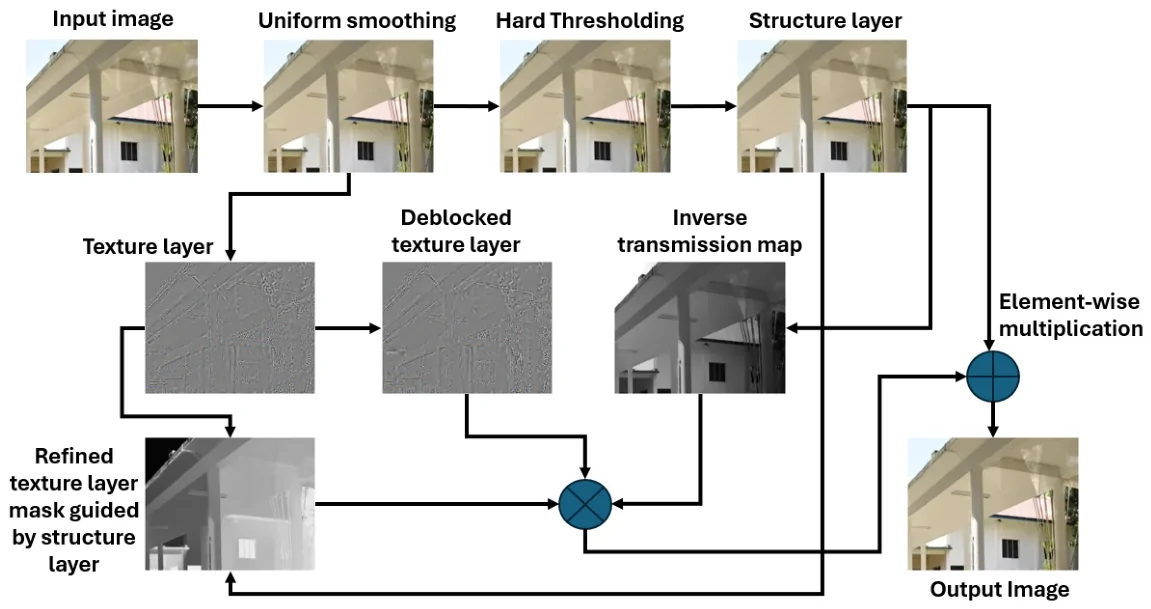
Overview of the proposed algorithm.

The main ingredient for the formation of our proposed model is the assumption that the reflection is transparent/out of focus in comparison to the background layer. This assumption can be formally interpreted as the following expression, as described in [25]:

(2) I=ωB+(1−ω)(κ∗R);

where *I* is the input reflection image, *B* is the background layer, *R* is the reflection layer, ω is the weight blending matrix, and κ is the blurring Gaussian kernel. The proposed model is further divided into five steps. Each step will be defined in detail in the subsequent sections of this paper. The overview of the proposed algorithm is shown in Figure 2.

### 2.1. Uniform Smoothing

Keeping in mind the assumption noted above, we examined the image smoothing technique in [41]:

(3) $$\min_{I_{st},G}{∥L\left(I_{st}\right)-L\left(I\right)∥}_{2}^{2}+\lambda C\left(G\right)+\beta {∥\nabla \left(I_{st}\right)-G∥}_{2}^{2},$$

where

(4) $$C\left(G\right)=C\left(H,V\right)=\#\{\left(i,j\right)||H_{i,j}|+|V_{i,j}|\neq 0\},$$

Equation (3) is solved by alternatively minimizing over $I_{st}$ and *G*. Sub-problems are given as follows. Sub-problem 1: Optimizing $I_{st}$ while keeping *G* fixed.

(5) $$\min_{I_{st}}{∥L\left(I_{st}\right)-L\left(I\right)∥}_{2}^{2}+\beta {∥\nabla \left(I_{st}\right)-G∥}_{2}^{2},$$

Equation (5) is quadratic and can be solved using FFT. But, according to the authors of [14], the FFT of the Laplacian fidelity term causes some color shifts in the resulting images. Therefore, the expression is optimized using an accelerated first-order gradient descent scheme, ADAM [42].

Sub-problem 2: Optimizing *G* while keeping $I_{st}$ fixed.

(6) $$\min_{G}{∥\nabla \left(I_{st}\right)-G∥}_{2}^{2}+\frac{\lambda }{\beta }C\left(G\right),$$

The above-defined smoothing approach globally attenuates the gradient magnitudes while keeping the structural similarity of the resulting image with the input image.

In Algorithm 1, *I* is the input image, i,j are the pixel indices, λ is the smoothing variable that controls the amount of smoothness, and β is the adaptive parameter that controls the similarity between the auxiliary variables (H,V) and their respective corresponding gradients $\left(\nabla_{x}I_{st},\nabla_{y}I_{st}\right)$. The range for λ used is [0.0005, 0.001] for quality results. $\beta_{\max}$ is the upper bound on β, at which the optimization terminates. G=(H,V) is the auxiliary vector field approximating the image gradient, with *H* and *V* its horizontal and vertical components, respectively. κ is the rate of convergence of the algorithm [41]. It is set within the range (1,2]. The default value is 2 for natural images.
**Algorithm 1** Uniform Smoothing using ADAM**Require:** *I*, λ, $\beta_{\max}$**Ensure:** $I_{st}$1:**Initialize:** $I_{st}\leftarrow I$, β←2λ, κ (1<κ≤2, κ=2 by default)2:**while** $\beta \leq \beta_{\max}$ **do**3:      **for** each pixel (i,j) **do**4:            Compute:$$\left(H_{i,j},V_{i,j}\right)=\left\{\begin{array}{cc} (0,0), & {\left(\nabla_{x}I_{st}^{i,j}\right)}^{2}+{\left(\nabla_{y}I_{st}^{i,j}\right)}^{2}\leq \frac{\lambda }{\beta }\\ \left(\nabla_{x}I_{st}^{i,j},\nabla_{y}I_{st}^{i,j}\right), & \mathrm{otherwise} \end{array}\right.$$5:      Compute gradient with respect to $I_{st}$:$$g=2L^{T}\left(L\left(I_{st}\right)-L\left(I\right)\right)+2\beta \nabla^{T}\left(\nabla I_{st}-G\right)$$6:      Update $I_{st}$ using an ADAM step with gradient *g*7:      β←κβ8:**return** $I_{st}$

### 2.2. Structure–Texture Decomposition

The resultant image of the uniform smoothing algorithm is then decomposed to extract the texture layer with minimized reflection and strong scene details. Since uniform smoothing smoothed out the reflections, the texture layer of the resultant is considerably less affected by the reflections. The texture layer extracted at this stage is then used to reintroduce the high-frequency components in the final output image. The structure–texture decomposition is achieved by using the Relative Total Variation-based structure–texture decomposition method proposed in [2]. The objective function of which can be expressed as:

(7) $$\min_{I_{s}}\sum_{i,j}{\left(I_{S_{i,j}}-I_{st}\right)}^{2}+\lambda *\left(\frac{D_{x_{i,j}}}{T_{x_{i,j}}+\epsilon }+\frac{D_{y_{i,j}}}{T_{y_{i,j}}+\epsilon }\right),$$

where the term ${\left(I_{S_{i,j}}-I_{st}\right)}^{2}$ is to ensure that the extracted structures are similar to the input image $I_{st}$. $\left(\frac{D_{x_{i,j}}}{T_{x_{i,j}}+\epsilon }+\frac{D_{y_{i,j}}}{T_{y_{i,j}}+\epsilon }\right)$ is the relative total variation, where ϵ is a small positive number to avoid division by zero. λ is the weight parameter. The minimization is done using half-quadratic splitting scheme, as was done in [2,13].

In Algorithm 2, $I_{st}$ is the resulting image of the uniform smoothing algorithm. p,q are pixel indices, with *q* ranging over the window R(p) centered at *p*. $I_{S}$ is the structure layer. $D_{x},D_{y}$ are the windowed total variation measures, which capture the total gradient energy within the window. $T_{x},T_{y}$ are the windowed inherent variation measures, which capture the net gradient within the window. $g_{p,q}$ is the spatial affinity weight, which decays with distance between *p* and *q* according to the scale parameter σ. σ controls the size of the window. λ controls the suppression of texture.

The decomposition is done in two stages. The first stage is after the uniform smoothing to get the texture layer, and the second stage is after hard thresholding to get the structure layer, which is then used to compute a mask for minimizing the reflections in the texture layer from the first stage.
**Algorithm 2** Structure–Texture Decomposition via Relative Total Variation**Require:** $I_{st}$, λ, σ, ϵ, *N***Ensure:** $I_{S}$, $I_{T}$1:**Initialize:** $I_{S}\leftarrow I_{st}$, $\sigma_{iter}\leftarrow \sigma$, λ←λ/22:**for** iter=1 to *N* **do**3:      Compute spatial affinity weights over each window R(p)$$g_{p,q}\propto \exp\left(-\frac{{\left(x_{p}-x_{q}\right)}^{2}+{\left(y_{p}-y_{q}\right)}^{2}}{2\sigma_{iter}^{2}}\right)$$4:      Compute windowed total variation:$$D_{x}\left(p\right)=\sum_{q\in R(p)}g_{p,q}\cdot \left|{\left(\partial_{x}I_{S}\right)}_{q}\right|,\;D_{y}\left(p\right)=\sum_{q\in R(p)}g_{p,q}\cdot \left|{\left(\partial_{y}I_{S}\right)}_{q}\right|$$5:      Compute windowed inherent variation:$$T_{x}\left(p\right)=\left|\sum_{q\in R(p)}g_{p,q}\cdot {\left(\partial_{x}I_{S}\right)}_{q}\right|,\;T_{y}\left(p\right)=\left|\sum_{q\in R(p)}g_{p,q}\cdot {\left(\partial_{y}I_{S}\right)}_{q}\right|$$6:      Update $I_{S}$ by solving:$$I_{S}\leftarrow \arg\min_{I_{S}}\sum_{i,j}{\left(S_{i,j}-I_{st_{i,j}}\right)}^{2}+\lambda \left(\frac{D_{x_{i,j}}}{T_{x_{i,j}}+\epsilon }+\frac{D_{y_{i,j}}}{T_{y_{i,j}}+\epsilon }\right)$$7:      $\sigma_{iter}\leftarrow \max\left(\sigma_{iter}/2,\;0.5\right)$8:**return** $I_{S}$

### 2.3. Hard Thresholding

We can re-formalize the image smoothing objective function for hard thresholding by removing the ${∥.∥}_{0}$ term and introducing the thresholding step directly into the objective function [7]. Although, instead of a fixed h, we adopt an adaptive spatially-varying threshold map. The proposed hard thresholding model can then be expressed as:

(8) $$\min_{Z^{c}}\frac{1}{2}{∥L\left(Z^{\left(c\right)}\right)-div\left(\delta_{\Lambda }⊙\nabla Y_{base}^{\left(c\right)}\right)∥}_{2}^{2}+\frac{\epsilon }{2}{∥Z^{\left(c\right)}-Y_{base}^{\left(c\right)}∥}_{2}^{2},$$

where *c* = 1, 2, 3 for each color channel; *L* = Laplacian; div = divergence operator; and $\delta_{\Lambda }$ = spatially-varying hard thresholding operator.

In Algorithm 3, $I_{ht}$ is the resulting image after hard thresholding, $I_{S}$ is the structure layer of the smoothed image, *S* is the resulting smoothed image after uniform smoothing, ϕ is the YCbCr color space, α is the blend coefficient, N is the min-max normalization, R is the range filter, and β,γ are the weighted thresholds. *h* is the gradient thresholding parameter that controls the weighted thresholds and is set at 0.05 to get the desirable results in the resulting image. The gradient thresholding parameter *h* was evaluated over h∈{1, 0.5, 0.1, 0.05} on the In-The-Wild dataset; nearly all metrics improve as *h* decreases and plateau quantitatively between h=0.1 and h=0.05, except for BRISQUE, which shows a small, consistent increase. Visual inspection revealed that h=0.1 introduces localized banding artifacts in flat, low-texture regions that h=0.05 avoids at no measurable metric cost, so h=0.05 was adopted as the final value.

Yang et al. [7] apply a single threshold *h* uniformly across the entire image. This method struggles when the image contains both bright and dark regions, as a threshold that works effectively for one region will over- or under-suppress the other. The proposed method instead computes two Otsu-based binary masks over the image and compares their pixel counts to determine whether the bright region or the dark region contains more pixels. Appropriate thresholds are then assigned to each region accordingly. This allows the degree of suppression to adapt across regions of mixed brightness within a single image, rather than being governed by a single fixed threshold value. The modified filtering separately addresses the dark and bright regions of an image using the mean/standard deviation statistics. The thresholding done in the YCbCr colorspace provides a better chance of removing the reflection without distorting the color balance of the image.
**Algorithm 3** Hard Thresholding**Input:** $I_{S},S$**Output:** $I_{ht}$1:Initialize $Y_{base}\leftarrow \phi \left(S\right),h=0.05$2:**Compute enhanced image and contrast map:**3:$I_{enh}\leftarrow \alpha G+\left(1-\alpha \right)N\left(H_{G}\right)$4:                                                      $A=R\;\left(N\;\left(L\;\left(\sum_{c=1}^{3}\left|Y_{base}^{\left(c\right)}\right|\right)\right)\right)$5:$\tau \leftarrow \mathrm{Otsu}(I_{enh})$6:$M_{1}\leftarrow 1_{\left\{I_{enh}>\tau \right\}},\;M_{2}\leftarrow 1_{\left\{I_{enh}\leq \tau \right\}}$7:$A_{1}\leftarrow A⊙M_{1},\;A_{2}\leftarrow A⊙M_{2}$8:**Auxiliary definitions:**9:$G\leftarrow \sum_{c=1}^{3}\left|I_{S}^{\left(c\right)}\right|$10:$H_{G}\leftarrow L\left(G\right)$11:**Threshold parameters:**12:$\beta \leftarrow 2h\sigma^{2}\left(A_{1}^{+}\right)+0.6h\mu \left(A_{1}^{+}\right),\;\gamma \leftarrow 0.5h\mu \left(A_{2}^{+}\right)+2h\sigma^{2}\left(A_{2}^{+}\right)$13:**Binary mask computation:**14:Calculate $s_{1}=\sum M_{1}$ **AND** $s_{2}=\sum M_{2}$15:**if** $s_{1}>s_{2}$ **then**16:      $\delta_{\Lambda }\leftarrow 1_{A_{1}>\gamma }\vee 1_{A_{2}>\beta }$17:**else**18:      $\delta_{\Lambda }\leftarrow 1_{A_{1}>\beta }\vee 1_{A_{2}>\gamma }$19:**Reconstruction:**20:$P\leftarrow L\left(\mathrm{div}\left(\delta_{\Lambda }⊙\nabla Y_{base}^{\left(c\right)}\right)\right)+\epsilon I_{st}$21:$K_{i,j}\leftarrow 2\left(\cos\left(i\pi /M\right)+\cos\left(j\pi /N\right)-2\right),\;0\leq i\leq M,\;0\leq j\leq N$22:$I_{ht}\left(i,j\right)\leftarrow F_{c}^{-1}\left({\left[F_{c}\left(P\right)\right]}_{i,j}/\left(K_{i,j}^{2}+\epsilon \right)\right)$23:**return** $I_{ht}$

### 2.4. Reflection and Artifact Removal

Once the structural layer of the resulting thresholded image and textural layer of the uniformly smoothed image are extracted, we need to remove the reflections and artifacts from the textural layer for the final output. For this reason, a mask is created that separates the regions of most of the scene details from the rest of the regions. The mask is created by applying a DCT-based filtering operation [4] and extracting the high-frequency coefficients. Initial estimation of the mask is obtained by thresholding the likelihood of high-frequency coefficients, where the likelihood function can be expressed as:

(9) $$Likelihood=\sum_{u,v}C_{u,v}^{2}-C_{1,1}^{2}-C_{1,2}^{2}-C_{2,1}^{2},$$

where *C* is the matrix of DCT coefficients of an 8 × 8 block of the input image; and *u*, *v* are the coefficient indices of 8 × 8 block. The DCT mask threshold was evaluated over {0.05, 0.1, 0.5} on the In-The-Wild dataset; full-reference metrics stay nearly flat, while no-reference metrics (BRISQUE, PIQE) degrade substantially at 0.5 due to over-smoothing, which was confirmed visually. The adopted value of 0.1 performs comparably to 0.05 across all metrics while remaining in this perceptually stable regime. Once the mask is created, we can keep the content of the regions with details while removing the content from the rest of the regions. Regions containing details also retain some reflections that can be removed by refining the mask. The mask is refined by applying a soft-matting technique to the structure layer. The soft-matting technique is inspired by the Dark Channel Prior estimation [43] and can be achieved by minimizing the following function:

(10) $$\min_{m}{\left(m-\hat{m}\right)}^{T}\left(m-\hat{m}\right)+\alpha m^{T}L_{s}m,$$

where *m* is the vector form of *M* (final mask estimation), $\hat{m}$ is the vector form of $\hat{M}$ (initial mask estimation), $L_{s}$ is the matting Laplacian matrix derived from extracted structure layer, and α is the regularization parameter set to $10^{5}$. The first term in the soft-matting function establishes consistency with the block-wise initial estimation of the mask, $\hat{M}$. The second term ensures that the refined mask is in alignment with the structural layer. Implementing the refined mask on the texture layer takes care of the rest of the reflections in the texture layer. Another benefit of this method is that, if any other artifacts like ringing, etc., are present in the image, they are removed as well.

### 2.5. Image Reconstruction

To reconstruct the final output image, we need to de-block the texture layer and adjust its contrast according to the structure layer. For de-blocking, the function is defined as:

(11) $$\min_{I_{T_{i}}^{d}}\left\{\sum_{i}{\left(I_{T_{i}}^{d}-I_{T_{i}}\right)}^{2}\right\}+\beta \sum_{i\in \eta }{\left(\nabla I_{T_{i}}^{d}\right)}^{2},$$

where $I_{T_{i}}$ is the texture layer at pixel index *i*. β is the weight term adapting the smoothness level. It was swept over {0.5, 0.7, 0.9}; higher values slightly improve full-reference metrics but worsen perceptual quality (BRISQUE, PIQE) by over-suppressing detail, so 0.7 was chosen as the best balance, consistent with visual inspection. η are the locations at the borders of 8 × 8 blocks. $I_{T_{i}}^{d}$ is the de-blocked texture layer. Once the texture layer is effectively de-blocked, the contrast of the texture layer must be adjusted to be in sync with the structural layer. This contrast adjustment is achieved by scaling the texture layer by the Dark Channel Prior estimation of the structure layer, as in [4,43]. The final texture layer can be estimated by the following expression:

(12) $$I_{T}^{e}=K*M*I_{T}^{d},$$

where *K* = Dark Channel Prior estimation of the structure layer, *M* is the refined mask, $I_{T}^{d}$ is the de-blocked texture layer, and $I_{T}^{e}$ is the contrast adjusted texture layer. The final background layer can be defined as:

(13) $$B=I_{S}+I_{T}^{e},$$

where $I_{S}$ is the final structure layer, $I_{T}^{e}$ is the final texture layer, and *B* is the final output of the proposed reflection removal algorithm.

### 2.6. Ablation and Pipeline Component Performance

To evaluate the contribution of each stage of the pipeline, we conducted a cumulative ablation on the In-The-Wild dataset (N=15), progressively adding each stage of the pipeline and assessing its output at each stage. The complete ablation study is provided in Table 1 for full-reference metrics (PSNR, MSE, SSIM, Global_NCC, Local_NCC, LMSE, SI) and no-reference metrics (BRISQUE, NIQE, PIQE) for each stage.

As shown in Table 1, the intermediate stages progress through a gradual decline in full-reference fidelity metrics (PSNR, SSIM, Global/Local NCC, SI) from uniform smoothing through the DCT-based mask estimation stage, with a small partial recovery on these same metrics at the soft-matting stage, while no-reference perceptual quality metrics (BRISQUE, NIQE, PIQE) fluctuate over the same span rather than following a monotonic trend: all three degrade at hard thresholding, improve sharply at the structure–texture decomposition stage—which achieves the best BRISQUE (31.81) and best PIQE (49.30) of any row—and then partially reverse this gain at the DCT-based mask estimation and soft-matting stages. This pattern indicates that the structure–texture decomposition stage in particular contributes a substantial perceptual-quality gain, though this gain is not fully retained through the subsequent DCT-masking and soft-matting stages, at a temporary cost to pixel-level fidelity relative to the ground truth—a cost that has yet to be fully compensated because these stages operate on a processed intermediate representation rather than reintroducing the original scene’s high-frequency content. We note that DCP-derived inverse-transmission weighting is applied from the DCT-based mask estimation stage onward, and the DCT-derived mask is progressively refined from a binary map (DCT stage) to a fully matted, tone-curve-adjusted soft mask (soft-matting stage).

The final reconstruction stage reverses this trend: PSNR improves by 0.61 dB, SSIM by 0.051, LMSE improves by 33%, and SI by 0.067 relative to the soft-matting stage, while no-reference perceptual metrics remain within a narrow range of their prior values (BRISQUE and NIQE both improve marginally; PIQE increases modestly). Notably, the DCT-based mask estimation and soft-matting stages already incorporate the DCP-derived inverse-transmission weighting and the fully refined soft mask, respectively; the sole distinction between the soft-matting and final reconstruction stages is the application of block-artifact de-blocking to the texture layer prior to recombination. This isolates texture de-blocking as the primary driver of the method’s full-reference accuracy gains, indicating that it is necessary to recover the fidelity given up by the intermediate processing stages without sacrificing the perceptual quality gains those stages achieve.

## 3. Results

The proposed algorithm was evaluated on various images and comparisons were made with both conventional and deep learning algorithms. The comparisons were based on the visual analysis of images and quantitative measures. The quality metrics include full-reference algorithms, such as PSNR (Peak Signal to Noise Ratio), SSIM (Structural Similarity Index), Global NCC (Normalized Cross-Correlation), Local NCC, SI (Structure Index), LMSE (Local Mean Squared Error), and MSE (Mean Squared Error). No-reference quality metrics, such as BRISQUE (Blind/Referenceless Image Spatial Quality Evaluator), NIQE (Naturalness Image Quality Evaluator), and PIQE (Perception based Image Quality Evaluator), are also used for comparison.

### 3.1. Datasets

The dataset used for our experiments was made publicly available by the ROSE Lab at Nanyang Technological University, Singapore, and is known as the Single-Image Reflection Removal (SIR2+) dataset [5]. It provides a large and highly diverse collection of mixed images, along with their corresponding ground truth background and reflection layers. To facilitate a comprehensive evaluation, the dataset is organized into three distinct parts: a controlled indoor set, an outdoor real-world set, and a purely In-The-Wild set.

#### 3.1.1. Indoor Dataset

The indoor subset consists of 40 scenes (20 solid objects, 20 postcards, 1200 images) and is designed to test in-focus reflections, out-of-focus blurs, and glass-induced ghosting [5]. It provides two key variations: focus variance across seven aperture settings from F11 to F32 (with 5 mm glass), where F32 gives the sharpest reflections and F11 the blurriest; and thickness variance using 3 mm, 5 mm, and 10 mm glass panels at fixed F32, where the 10 mm and 3 mm panels produce the largest and smallest spatial shifts, respectively, which allowed us to assess texture and structure preservation.

#### 3.1.2. Outdoor Dataset

The outdoor subset moves to real-world scenes with complex reflectances—cars, foliage, and windows—captured across varying distances and locations (residential halls, gardens, and lecture rooms) under diverse natural lighting, from direct sunlight to twilight [5]. It comprises 100 triplets, totaling 300 images.

#### 3.1.3. In-The-Wild Dataset

Finally, the In-The-Wild subset tests our method against irregular real-world properties like non-uniform glass curvatures and color tints, drawn from practical settings such as showcases, windows, and windshields [5]. This serves as the ultimate benchmark for evaluating generalization to completely uncontrolled imaging conditions.

### 3.2. Error Metrics

The SIR2+ dataset was used to evaluate the performance of the proposed algorithm against representative single-image reflection removal methods, including LB14 [13], WS16 [29], NR17 [25], YW19 [7], CoRRN [44], ZN18 [36], YD18 [37], ERRNet [45], SIRRBL [46], and IBCLN [47], for both quantitative accuracy (with respect to ground truth) and visual quality. A comprehensive suite of full-reference and no-reference evaluation metrics is utilized for this purpose, which is detailed below.

#### 3.2.1. Full-Reference Metrics

MSE, which measures the global pixel-wise intensity variance, provides a broad measure of reflection suppression across the entire image. Complementing this global assessment, LMSE localizes the evaluation to specific image regions and captures the algorithm’s effectiveness in removing small-scale imperfections that global metrics might overlook. Together with PSNR, which calculates the ratio between the maximum possible clean signal power and the residual distortions, these metrics provide a comprehensive picture of pixel-level fidelity—higher PSNR values confirm that the framework has successfully suppressed the overarching reflection intensity layer while preserving background signal strength.

Expanding from pixel-level fidelity to structural integrity, SSIM evaluates the similarity between the restored and ground truth images using luminance, contrast, and structure components. By modeling the human visual system with high sensitivity to structural distortion, SSIM ensures that the reflection removal process does not inadvertently smooth away or tear the underlying background contours. Taking structural assessment one step further, SI discards luminance and contrast to focus exclusively on object boundaries, allowing it to specifically assess whether the algorithm successfully separates the background from the sharp, intersecting structural lines of an in-focus reflection.

To verify spatial alignment and texture preservation, Global NCC measures the macro-level linear correlation between the restored image and ground truth, confirming overall spatial and directional alignment. Similarly, Local NCC performs this correlation within localized regions, ensuring that high-frequency textures are preserved within areas previously obstructed by reflections. Together, they provide both coarse and fine-grained validation that our algorithm maintains strict spatial and directional alignment throughout the restoration process.

#### 3.2.2. No-Reference Metrics

For scenes where ground truth is unavailable, we employ three complementary no-reference perceptual metrics. BRISQUE analyzes losses in naturalness by quantifying processing anomalies such as artificial blur or ringing artifacts, with lower scores validating a more natural, artifact-free output. Complementing this, NIQE measures the statistical distance from pristine natural scenes; given that reflections inherently disrupt native image statistics, a superior NIQE score (lowest value) proves that the reflection-suppressed image successfully conforms to the expected properties of a glass-free environment. Finally, PIQE evaluates localized visual distortion by analyzing the block-wise noticeability of artifacts like blur and blockiness, with low scores confirming that reflection suppression does not leave behind visually jarring patches or geometric cloudiness. Together, these no-reference metrics provide a comprehensive assessment of perceptual fidelity across varying degrees of artifact visibility.

### 3.3. Evaluations

**Figure 3:**
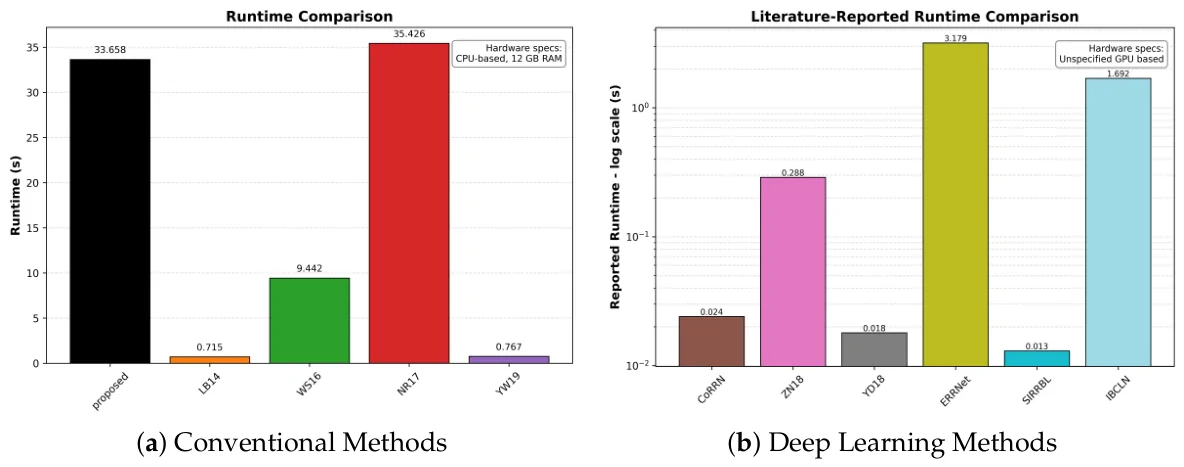
(**a**) Conventional methods with their runtime computed on CPU-based machine. (**b**) Deep learning methods with their runtime computed on unspecified GPU, reported from the literature [5].

Figure 3 compares runtime across methods. The proposed method and the traditional baselines (LB14, WS16, NR17, YW19) were measured on identical CPU-based hardware (11th Gen Intel Core i5-1135G7 @ 2.40 GHz, base clock 2.42 GHz, 12 GB RAM), while the deep learning methods (CoRRN, ZN18, YD18, ERRNet, SIRRBL, IBCLN) report literature-reported runtimes obtained on unspecified GPU hardware, and the two groups are therefore not directly comparable on speed alone.

Under this CPU-only setting, the proposed method (33.658 s) is comparable to NR17 (35.426 s) and substantially slower than LB14, WS16, and YW19, reflecting the added cost of the L0 gradient-based smoothing stage. While the GPU-accelerated deep learning methods report substantially lower runtimes, they depend on specialized hardware, large-scale training data, and trained model weights that the proposed method does not require, allowing it to be deployed on general-purpose hardware without GPU acceleration.

#### 3.3.1. Quantitative Performance

The Table 2, Table 3, Table 4, Table 5, Table 6 and Table 7 show the overall quantitative performance of the indoor, outdoor, and In-The-Wild datasets using the error metrics above.

Table 2, Table 3, Table 4, Table 5, Table 6 and Table 7 report the quantitative comparisons across the indoor, outdoor, and In-The-Wild datasets for non-learning and deep learning methods. We analyze the results by separating the No-Reference (NR) perceptual metrics from the Full-Reference (FR) reconstruction metrics, as they reflect fundamentally different optimization objectives.

#### 3.3.2. Reconstruction Fidelity

**Figure 4:**
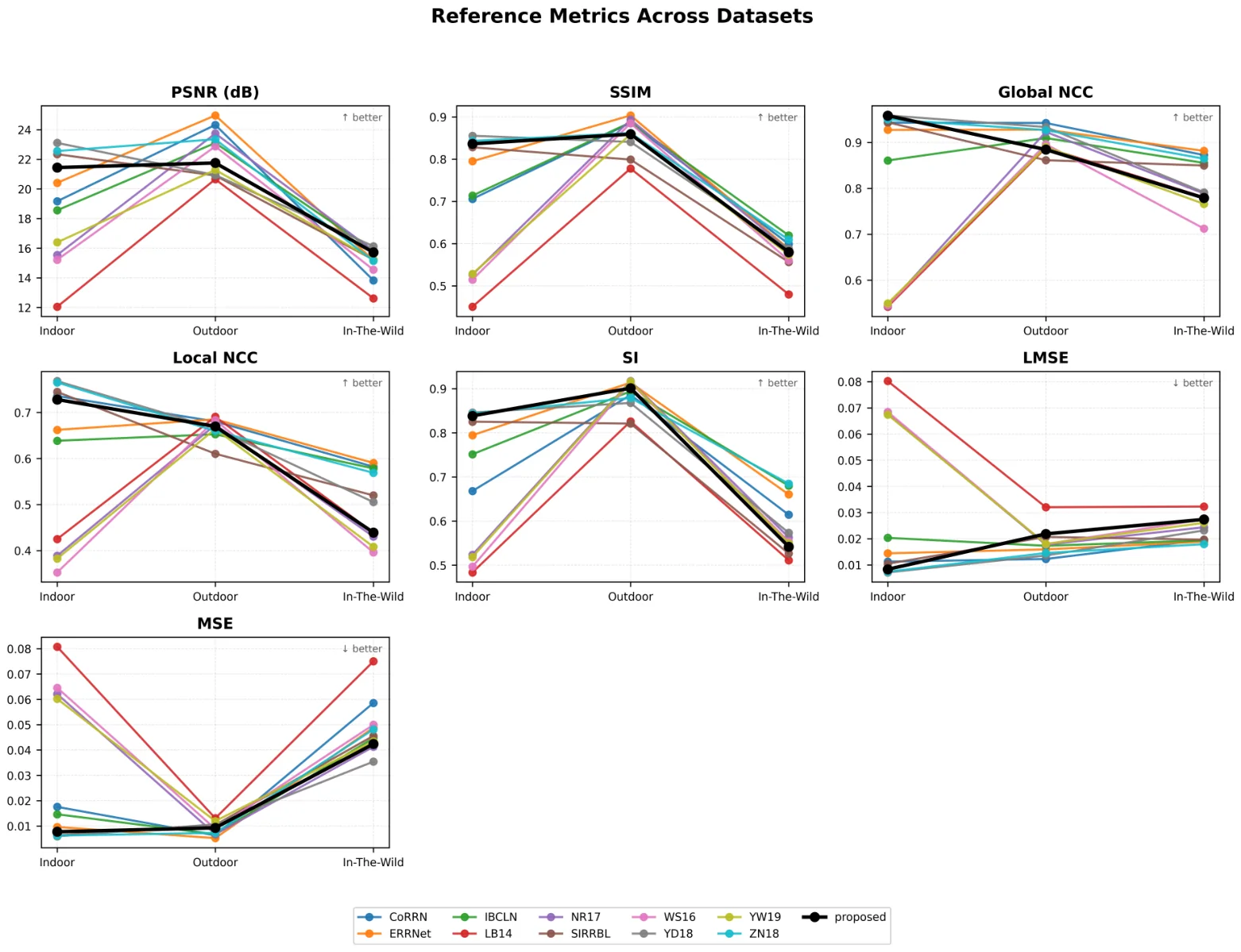
The average metric values of the proposed method against all the competitive methods across all datasets for reference metrics.

In terms of full-reference (FR) metrics, our method exhibits distinct domain-dependent behavior across the evaluated datasets, as summarized in Figure 4 and detailed in Table 2, Table 3, Table 4, Table 5, Table 6 and Table 7. On the indoor dataset, it achieves a competitive PSNR (21.448) and SSIM (0.836), placing it in the mid-tier among deep learning methods and superior to all traditional baselines; it performs comparably to ZN18 (22.560) and SIRRBL (22.347), while trailing the top-performing YD18 [37] by a margin of approximately 1.66 dB. On the outdoor dataset, our performance improves slightly, yielding a PSNR of 21.758, which surpasses deep learning methods such as YD18 (20.938) and SIRRBL (20.909), while maintaining a second-best SI of 0.901 among the deep learning methods.

On the more challenging In-The-Wild dataset, the method shows mixed performance relative to the deep learning baselines. It achieves the third-highest PSNR (15.745) among the deep learning models, behind YD18 (16.134) and IBCLN (15.841), while its SSIM (0.569) trails most competitors, ranking above only SIRRBL (0.556). Local structural fidelity is similarly limited on this unconstrained dataset: its Local NCC (0.402) falls below all deep learning baselines, and its LMSE (0.027) is the highest (worst) among the deep learning methods evaluated, indicating that the method’s structural and localized fidelity is comparatively weaker under unconstrained, real-world conditions than in the controlled indoor and outdoor settings.

Figure 4 presents these full-reference trends (PSNR, SSIM, Global NCC, Local NCC, SI, LMSE, MSE) across all three datasets for every evaluated method. Across most metrics, the proposed method tracks closely with the competing methods on the indoor and outdoor datasets, before its relative standing declines on the In-The-Wild dataset, particularly for Local NCC and LMSE, consistent with the trend described above.

#### 3.3.3. Perceptual Quality

**Figure 5:**
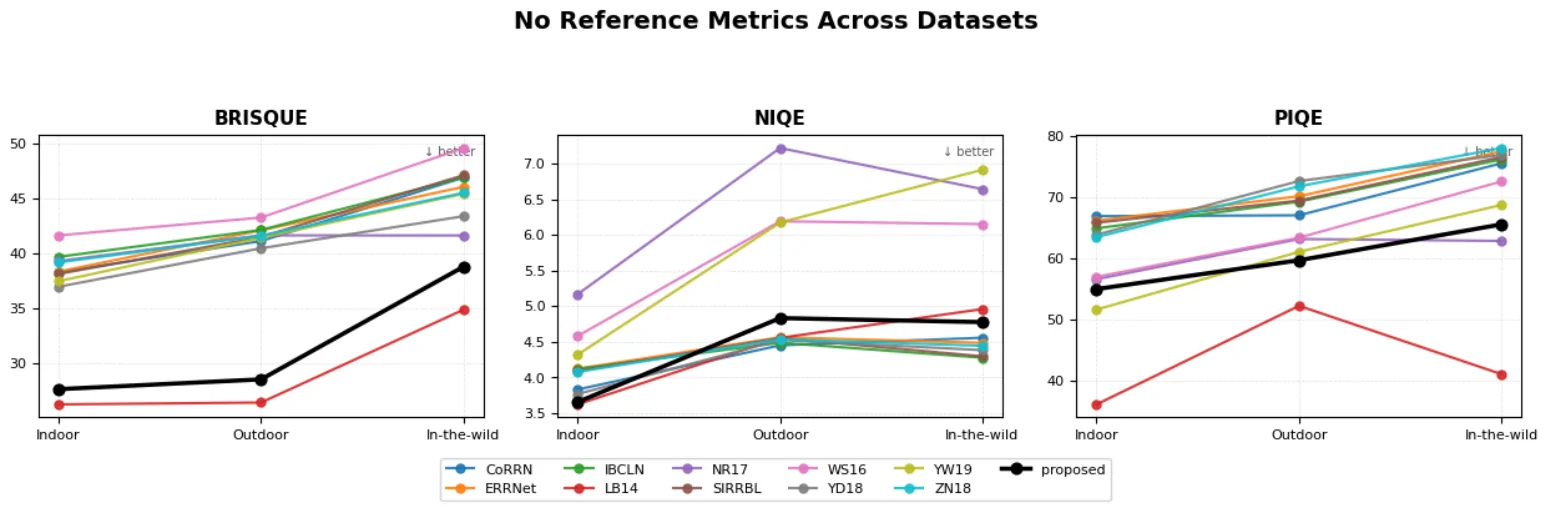
The average metric values of the proposed method against all the competitive methods across all datasets for no-reference metrics.

Our proposed method demonstrates consistent and competitive performance across all NR metrics among the non-learning methods, as shown in Figure 5 and detailed in Table 2, Table 3, Table 4, Table 5, Table 6 and Table 7. On the indoor dataset, it achieves the lowest BRISQUE (27.584) and PIQE (55.019) among the deep learning-based competitors, while among non-learning methods it ranks second-best in BRISQUE and third-best in PIQE. This trend persists on the outdoor dataset, where our method secures the best BRISQUE (28.478) and PIQE (59.719) among the deep learning methods, substantially surpassing competitor models such as YD18 (BRISQUE: 40.438; PIQE: 72.656) and ERRNet (42.082; 70.172); comparing the proposed method with non-learning ones, it ranks second-best for both metrics. On the In-The-Wild dataset, despite the increased scene complexity, our method achieves the lowest BRISQUE (34.189) among all competing methods, both non-learning and deep-learning, and secures the lowest PIQE (59.517) among the deep learning methods, while ranking second-best in PIQE among the non-learning methods, behind LB14 (41.129). These results indicate that our approach effectively suppresses visual artifacts and ringing, delivering outputs with strong naturalness that generalize well across diverse domains.

One exception is observed in the outdoor and In-The-Wild NIQE score, where our method records the highest (worst) value among the deep learning methods (4.833 and 5.789, respectively), though it remains the second-best NIQE score among the non-learning methods. We attribute this to NIQE’s reliance on fixed Natural Scene Statistics (NSS) priors that are particularly sensitive to uniform regions (e.g., skies and vegetation). Since our method preserves fine textural variations rather than imposing strong smoothing, it deviates from these statistical expectations, resulting in a higher NIQE penalty relative to the deep learning baselines, despite its clear advantages in the human-correlated BRISQUE and PIQE scores.

#### 3.3.4. Aperture Variation (F-Variance)

We assess the extreme aperture settings provided in the dataset: F11, which produces the greatest blur in the reflection layer, and F32, which yields the sharpest reflection. As reported in Table 8 and Table 9, our proposed method demonstrates notable robustness across this spectrum; rather than degrading under variation, it shows a slight improvement in reconstruction fidelity under the clearer scene conditions of F32, with PSNR climbing from 21.234 under F11 to 21.487 under F32. In contrast, competing deep learning baselines show clearer sensitivity to these aperture shifts, with several models suffering performance degradation when moving to the sharper structural profiles of the F32 setting. For instance, from F11 to F32, CoRRN’s PSNR drops from 19.281 to 18.905, while ZN18 experiences a drop from 22.777 to 22.392. Our method also consistently secures a top-three overall ranking in BRISQUE, NIQE, and PIQE across both aperture conditions, ranking first among the deep learning models evaluated, indicating that its perceptual advantages remain consistent across varying depth-of-field conditions.

#### 3.3.5. Glass Thickness Variation (T-Variance)

We next evaluate the extreme thickness configurations provided in the dataset: T3 (3 mm), which produces the smallest spatial shift between the reflected and transmitted layers, and T10 (10 mm), which produces the largest spatial shift. This increasing misalignment poses a significant registration challenge for reflection removal.

As shown in Table 10 and Table 11, our method proves consistently stable across this spatial disparity; rather than degrading under the larger separation, SSIM improves slightly from 0.831 to 0.834 from T3 to T10, while PSNR remains largely stable, decreasing marginally from 21.485 to 21.440. By contrast, competing deep learning models show clearer sensitivity to the increased spatial offset, with several frameworks suffering more pronounced performance drops from T3 to T10. For instance, CoRRN [44] experiences a PSNR degradation from 19.251 to 19.001, while SIRRBL [46] and ZN18 [36] decline from 22.309 to 22.174 and 22.478 to 22.372, respectively. Our method also maintains a top-three overall ranking under the demanding T10 condition, achieving the best BRISQUE (27.544), NIQE (3.522), and PIQE (54.851) scores among the deep learning models evaluated. This consistent stability across varying spatial offsets underscores the reliability of our approach for uncalibrated, real-world scenarios.

### 3.4. Qualitative Performance

**Figure 6:**
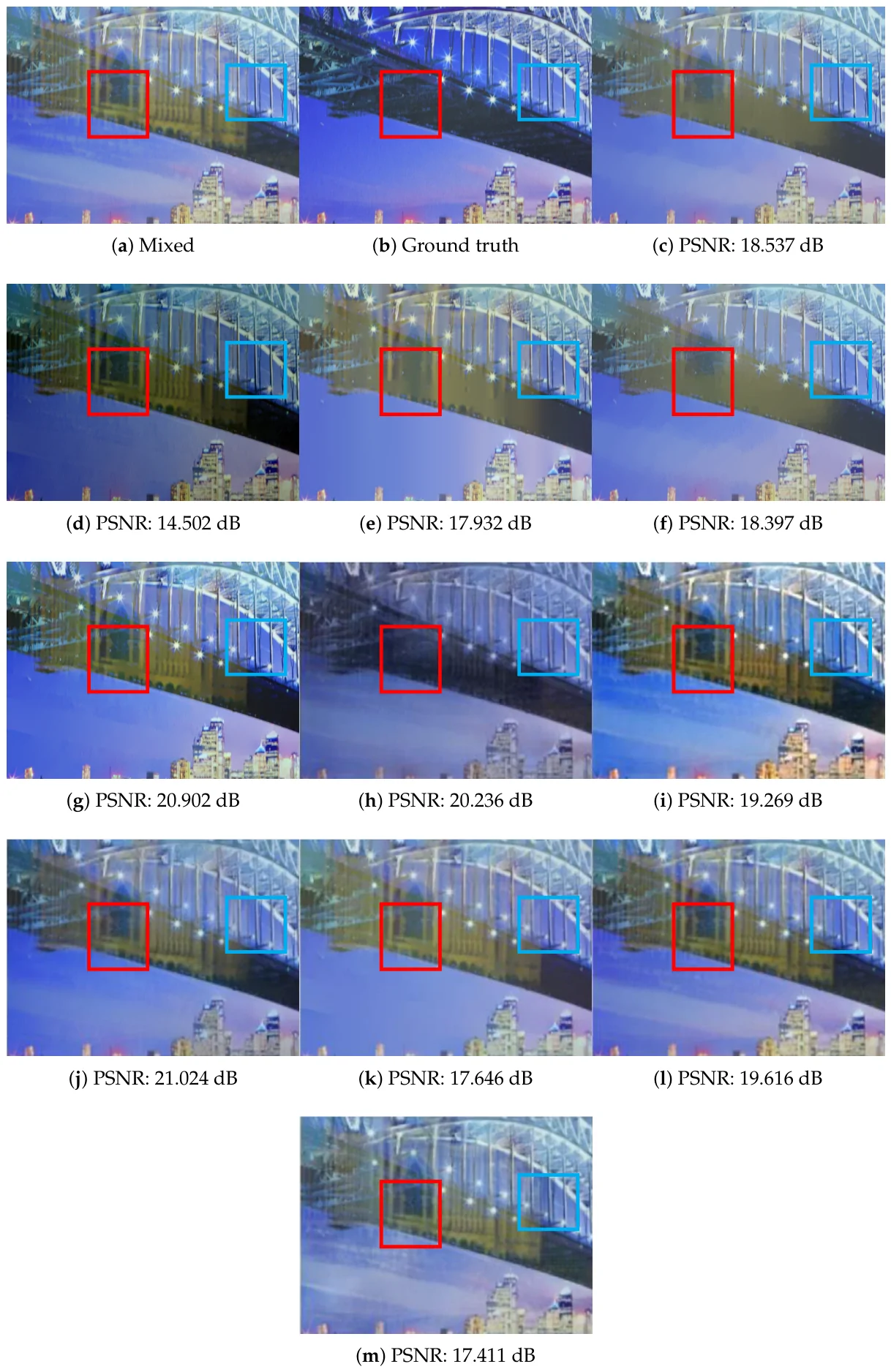
Qualitative comparison of proposed method with the competitive non-learning and deep learning methods on indoor dataset: (**a**) Mixture image, (**b**) Ground truth, (**c**) Proposed, (**d**) LB14 [13], (**e**) WS16 [29], (**f**) NR17 [25], (**g**) YW19 [7], (**h**) CoRRN [44], (**i**) ZN18 [36], (**j**) YD18 [37], (**k**) ERRNet [45], (**l**) SIRRBL [46], (**m**) IBCLN [47]. Red and blue boxes indicate the two regions of interest (ROIs) selected for magnified comparison across all methods.

**Figure 7:**
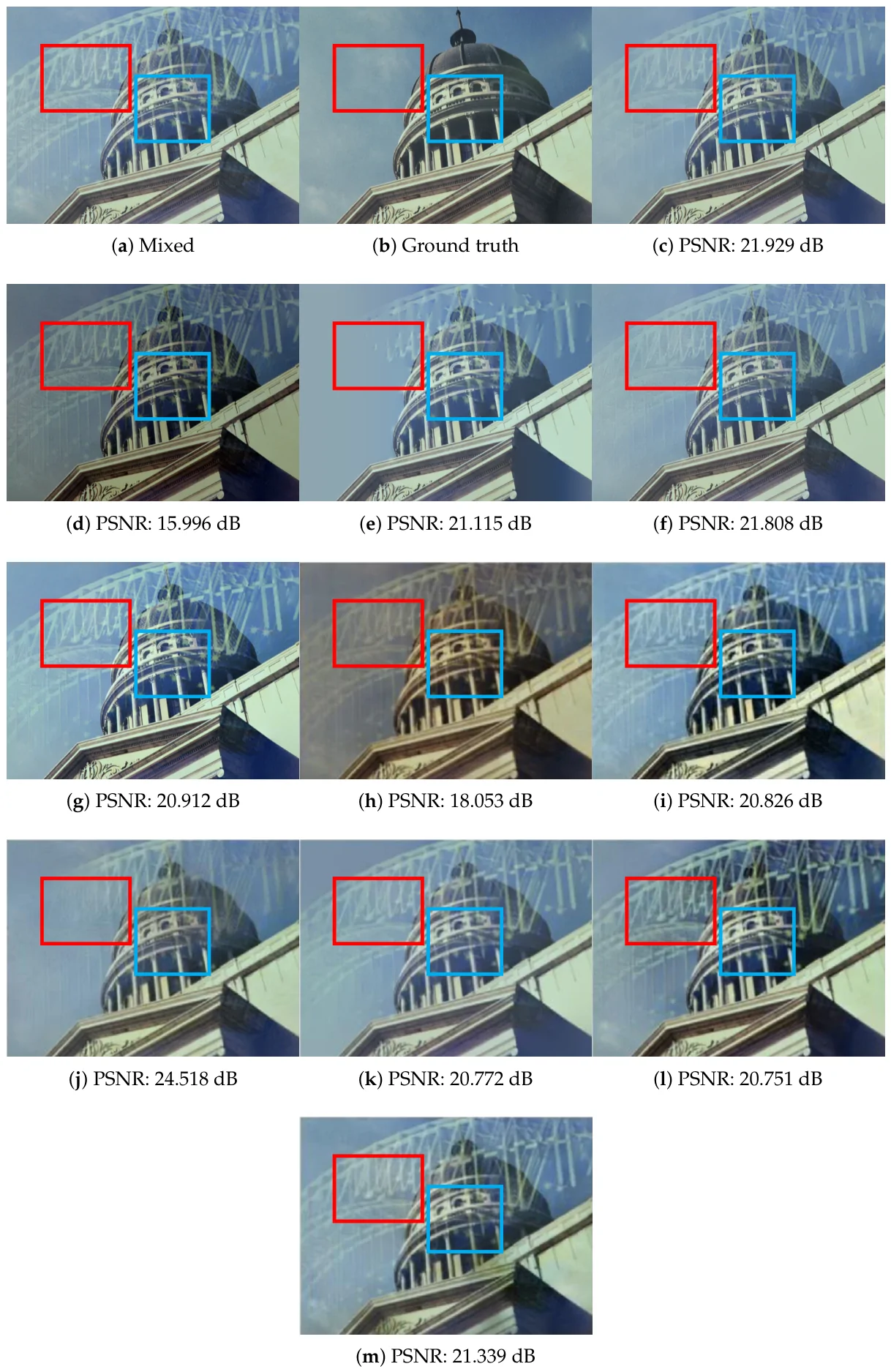
Qualitative comparison of proposed method with the competitive non-learning and deep learning methods on indoor dataset: (**a**) Mixture image, (**b**) Ground truth, (**c**) Proposed, (**d**) LB14 [13], (**e**) WS16 [29], (**f**) NR17 [25], (**g**) YW19 [7], (**h**) CoRRN [44], (**i**) ZN18 [36], (**j**) YD18 [37], (**k**) ERRNet [45], (**l**) SIRRBL [46], (**m**) IBCLN [47]. Red and blue boxes indicate the two regions of interest (ROIs) selected for magnified comparison across all methods.

**Figure 8:**
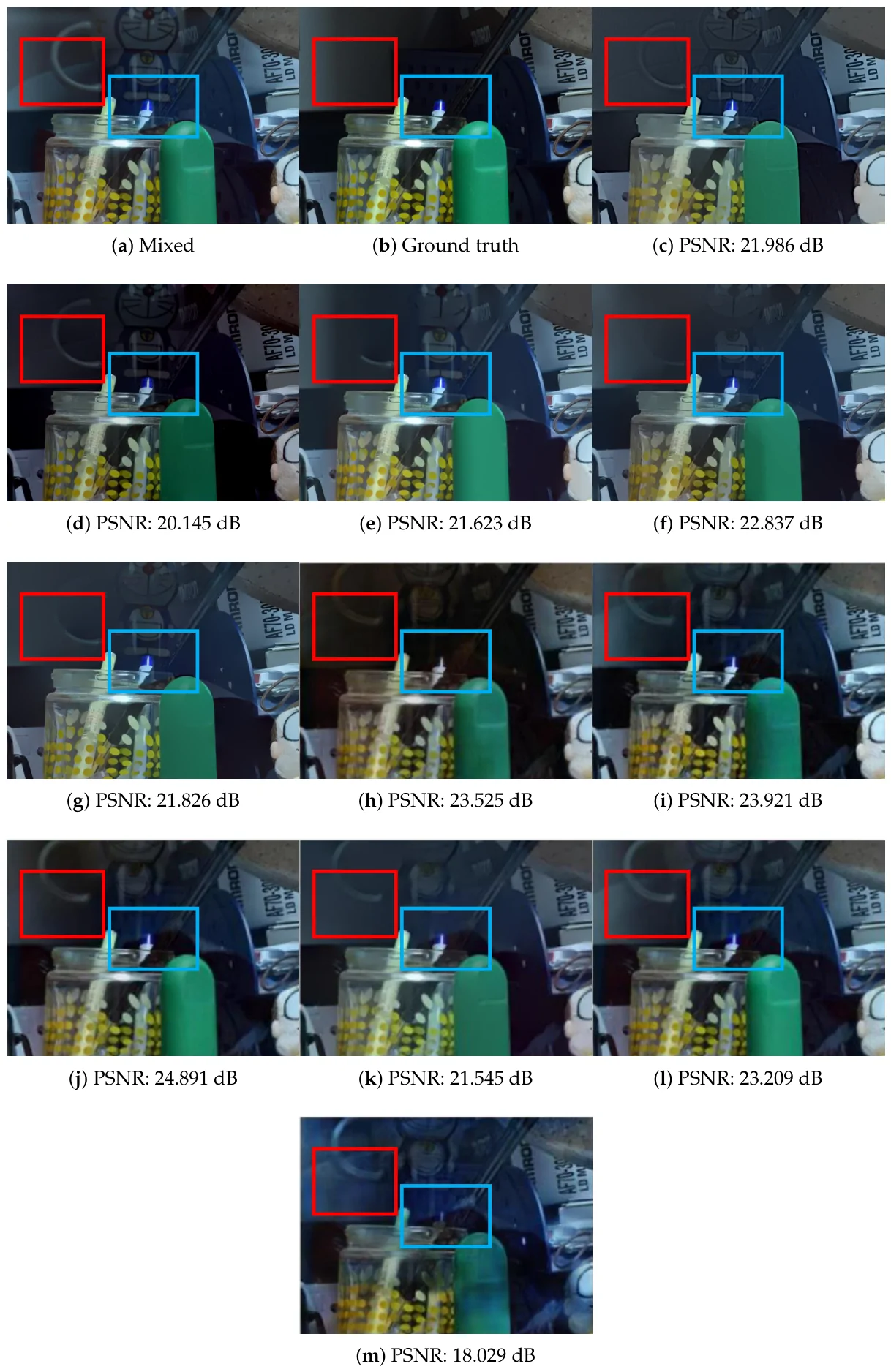
Qualitative comparison of proposed method with the competitive non-learning and deep learning methods on indoor dataset: (**a**) Mixture image, (**b**) Ground truth, (**c**) Proposed, (**d**) LB14 [13], (**e**) WS16 [29], (**f**) NR17 [25], (**g**) YW19 [7], (**h**) CoRRN [44], (**i**) ZN18 [36], (**j**) YD18 [37], (**k**) ERRNet [45], (**l**) SIRRBL [46], (**m**) IBCLN [47]. Red and blue boxes indicate the two regions of interest (ROIs) selected for magnified comparison across all methods.

To complement the quantitative results, we present qualitative comparisons on controlled indoor scenes in Figure 6, Figure 7 and Figure 8, containing strong reflections and well-defined structural content, including a bridge scene with reflective streaks, a dome with fine geometric details, and a cluttered object scene captured under low illumination. In Figure 6, which illustrates results from a bridge arch and its sky gradient, the methods are tested under severe reflection interference and global illumination challenges. Under sharp, vertical reflection grid lines over the bridge arch, conventional models LB14 [13] and YW19 [7] and deep learning models ERRNet [45] and IBCLN [47] struggle to isolate the background, while the deep learning method CoRRN [44] dims the reflection but introduces noticeable structural blurring; in contrast, the proposed method substantially suppresses the reflection layer. When evaluating the right-hand sky gradient and deck lights for artifact suppression, the conventional method LB14 [13] creates an unnatural dark exposure boundary, while methods WS16 [29] and NR17 [25] cause noticeable color washout. Furthermore, the deep learning framework ZN18 [36] produces a synthetic haze, while methods YD18 [37] and SIRRBL [46] generate visible horizontal banding noise across the smooth evening sky. The proposed method avoids most of these secondary distortions, maintaining a largely clear background gradient with minimal bleeding around the point lights. Figure 7 illustrates results from a dome’s peak and its drum columns, which were used to assess smooth background gradients and overlapping structural interference. Across the upper sky surrounding the dome’s peak, sweeping cursive-like reflection streaks heavily occlude the mixed input. Baselines NR17 [25], YW19 [7], ZN18 [36], ERRNet [45], SIRRBL [46], and IBCLN [47] largely fail to remove these streaks, while LB14 [13] noticeably darkens the region, WS16 [29] smears the reflection with a foggy distortion, and CoRRN [44] shifts the sky toward an unnatural sepia tone. The proposed method reduces these artifacts while largely maintaining the naturalness of the blue sky. Where diagonal reflection lines cross directly over the sharp vertical pillars of the dome’s drum, baselines NR17 [25], YW19 [7], ZN18 [36], ERRNet [45], and IBCLN [47] preserve much of the reflection over the pillars, and deep learning models CoRRN [44] and YD18 [37] introduce structural blurring that softens the sharp vertical lines despite suppressing reflection intensity. The proposed method handles this challenge comparatively well, suppressing the overlaying artifacts while largely preserving architectural edges and high-contrast details. Figure 8 illustrates results from a low-contrast dark background and a glass bottle opening, which were used to evaluate faint artifact suppression and edge-sharpness retention. Testing the upper-left dark background zone, a prominent curved white reflection loop sits noticeably in the dark space of the mixed input. Baselines NR17 [25], YW19 [7], ZN18 [36], YD18 [37], ERRNet [45], and SIRRBL [46] largely fail to attenuate this ghosting pattern, while LB14 [13] masks it by over-darkening the entire zone, WS16 [29] creates a blocky gradient distortion, and CoRRN [44] triggers noticeable contrast collapse that leaves the background muddy. The proposed method reduces this faint reflection with limited residual artifacts. Finally, near the high-detail glass bottle opening and protruding straw element where reflection layers cut through fine geometric contours, baselines LB14 [13], NR17 [25], YW19 [7], ERRNet [45], and SIRRBL [46] leave noticeable residual artifacts that distort the shape of the glass rim. Meanwhile, deep learning methods CoRRN [44] and IBCLN [47] introduce noticeable blurring and color artifacts, with IBCLN [47] introducing an unnatural blue haze that softens the foreground boundaries. The proposed method maintains the structural details of the background objects while reducing overlapping reflections to a considerable extent.

While the proposed framework exhibits minor trade-offs under extreme conditions, these results suggest that the proposed method produces visually plausible restorations, performing competitively with, and in several cases favorably against, both traditional non-learning benchmarks and deep networks under diverse real-world imaging conditions.

**Figure 9:**
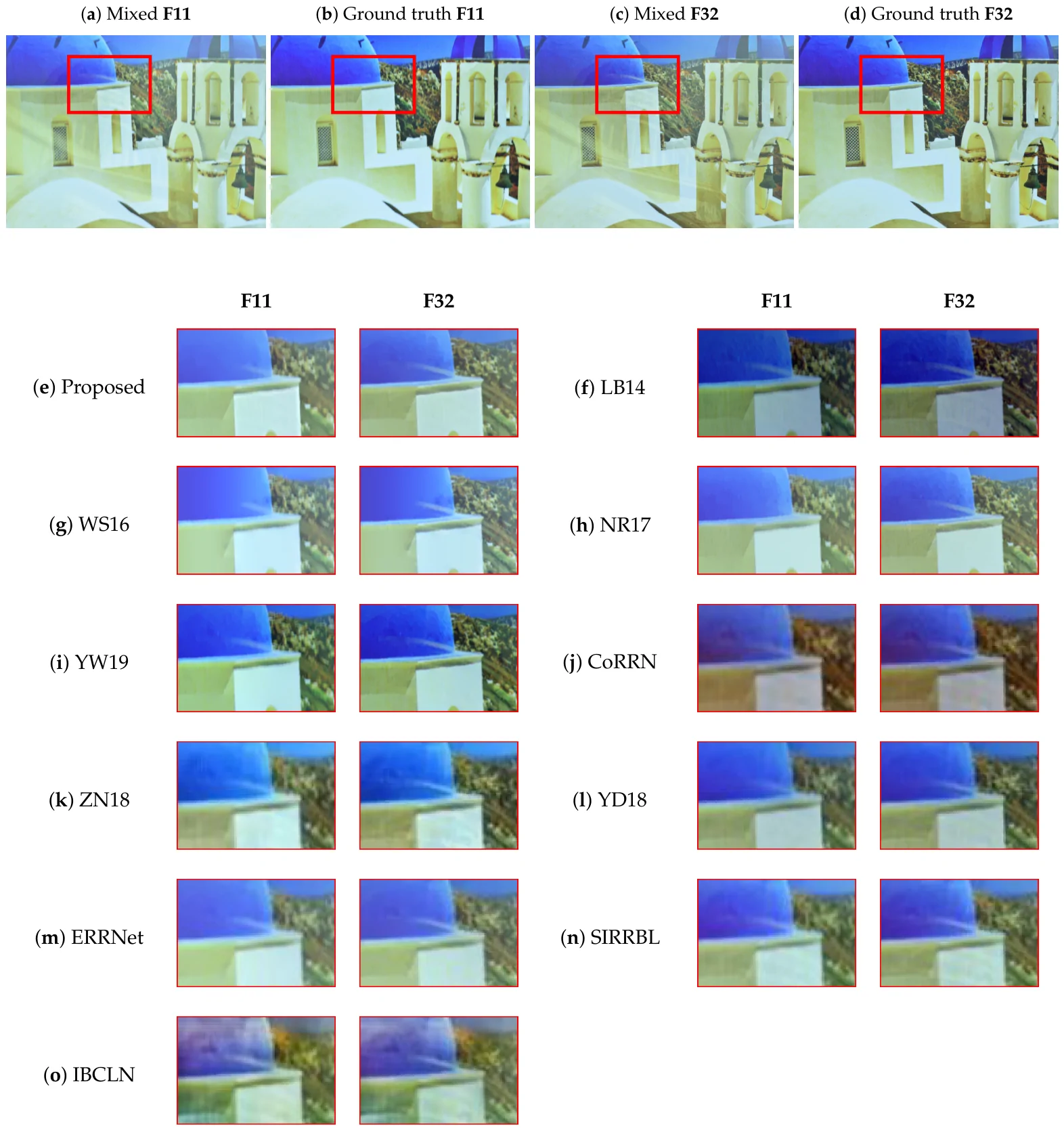
Qualitative comparison of competitive methods on extreme focus conditions of indoor dataset. Competing methods introduce a visible color cast over the wall and sky, whereas the proposed method preserves the true color balance and the roofline boundary. Red boxes indicate the region of interest (ROI) selected for magnified comparison across all methods.

**Figure 10:**
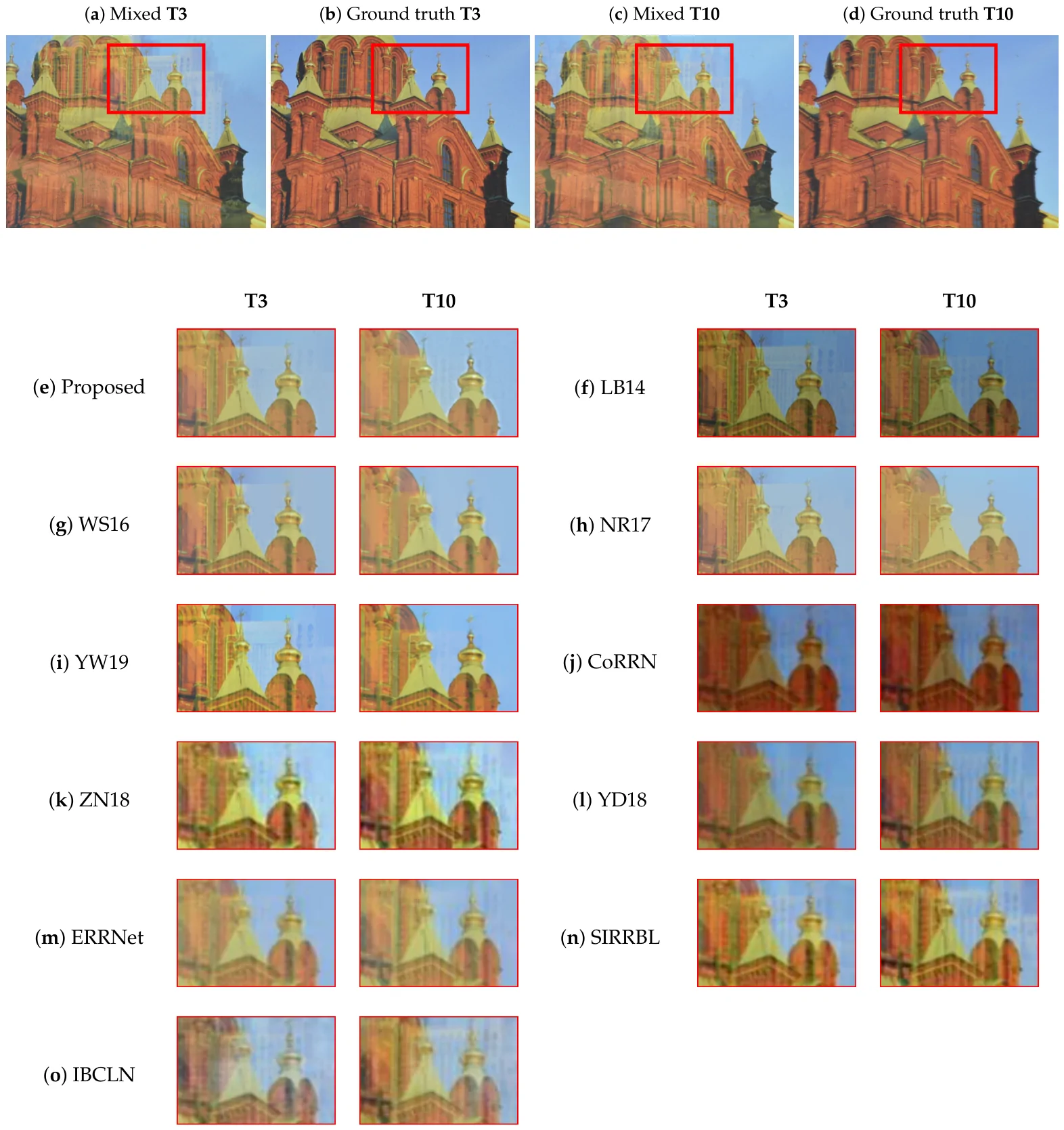
Qualitative comparison of competitive methods on extreme thickness conditions of indoor dataset. Competing methods retain a visible blue-tinted reflection haze that obscures fine structural detail on the domes, whereas the proposed method removes the reflection while preserving this detail. Red boxes indicate the region of interest (ROI) selected for magnified comparison across all methods.

To further assess robustness under extreme imaging conditions, we present qualitative comparisons for the aperture (F11/F32) and glass-thickness (T3/T10) subsets of the indoor dataset in Figure 9 and Figure 10, complementing the quantitative robustness analysis. Figure 9 illustrates results using a rooftop and sky region under narrow (F11) and wide (F32) aperture settings, where reflection intensity and blur vary with the amount of light entering the lens. The competing methods introduce a visible color cast over the wall and sky under both settings: LB14 [13] shows a pronounced warm cast that intensifies from F11 to F32, NR17 [25] and CoRRN [44] exhibit a strong yellow tint across the wall, YD18 [37] and SIRRBL [46] retain a milder but visible warm cast, and IBCLN [47] introduces a substantial purple color distortion across both apertures. WS16 [29], YW19 [7], ZN18 [36], and ERRNet [45] perform comparatively well, closely matching the ground truth color balance. The proposed method preserves a color balance and roofline boundary close to the ground truth across both aperture settings, with no noticeable degradation between F11 and F32. Figure 10 illustrates results from a domed structure under thin (T3) and thick (T10) glass conditions, where increased glass thickness typically strengthens the reflection layer. The competing methods retain a visible blue-tinted reflection haze that obscures fine structural detail on the domes: LB14 [13] produces a heavily darkened sky that worsens noticeably at T10, CoRRN [44] and IBCLN [47] exhibit a persistent magenta-toned reflection haze across both thicknesses, and ZN18 [36], YD18 [37], ERRNet [45], and SIRRBL [46] retain a milder blue-tinted haze with some loss of edge sharpness on the dome structures. The proposed method reduces this reflection haze while preserving fine structural detail on the domes across both glass-thickness levels. Taken together, these qualitative results suggest that the proposed method maintains stable performance across both extreme aperture and extreme glass-thickness conditions, complementing the quantitative robustness results reported in Table 8, Table 9, Table 10 and Table 11.

**Figure 11:**
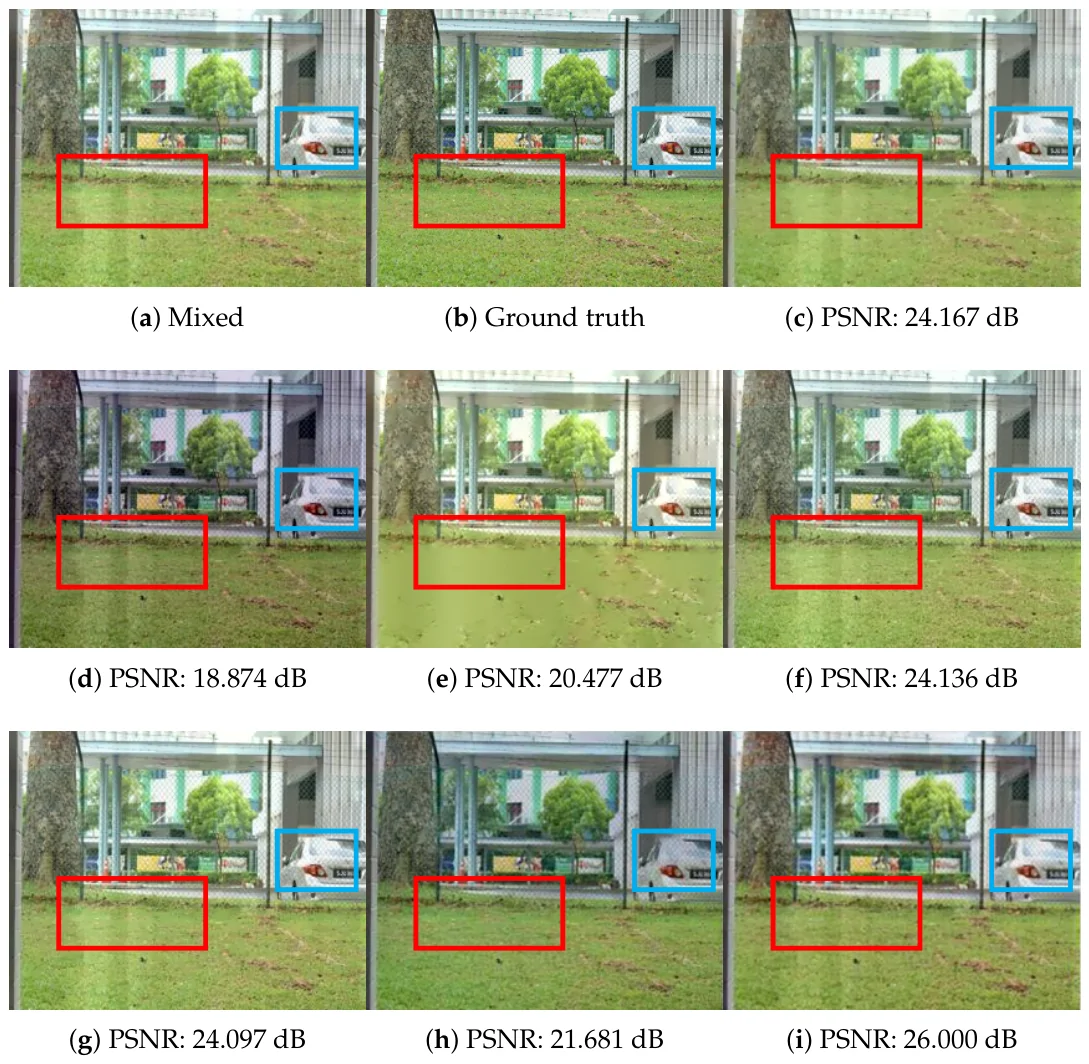

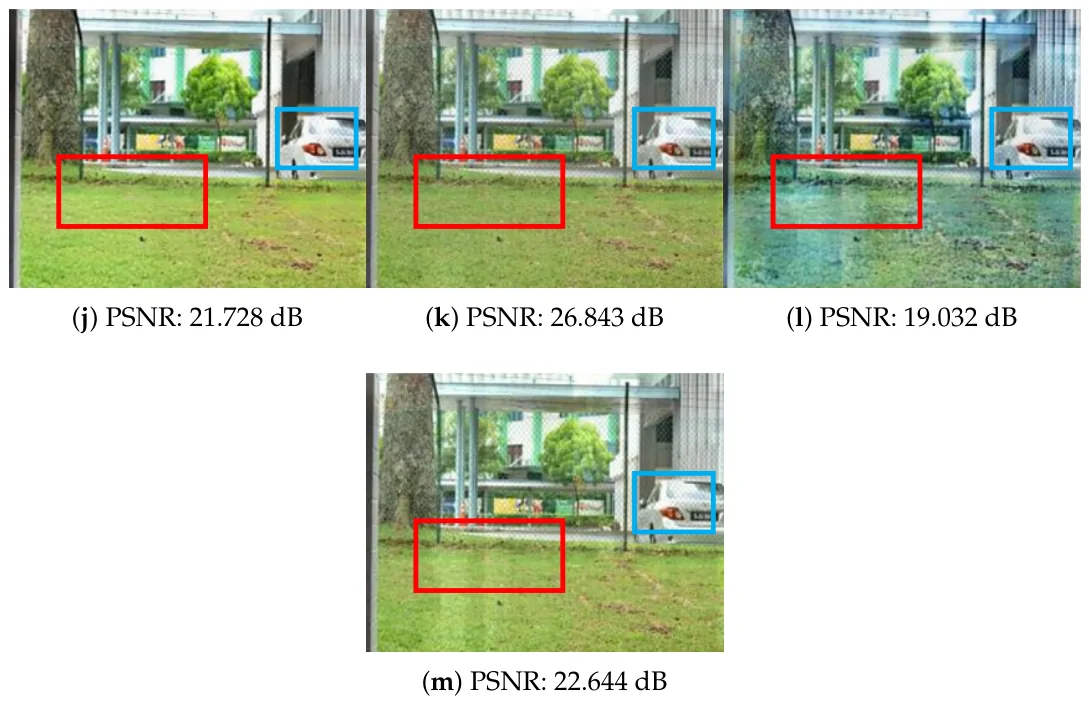
Qualitative comparison of proposed method with competitive non-learning and deep learning methods on outdoor dataset: (**a**) Mixture image, (**b**) Ground truth, (**c**) Proposed, (**d**) LB14 [13], (**e**) WS16 [29], (**f**) NR17 [25], (**g**) YW19 [7], (**h**) CoRRN [44], (**i**) ZN18 [36], (**j**) YD18 [37], (**k**) ERRNet [45], (**l**) SIRRBL [46], (**m**) IBCLN [47]. Red and blue boxes indicate the two regions of interest (ROIs) selected for magnified comparison across all methods.

**Figure 12:**
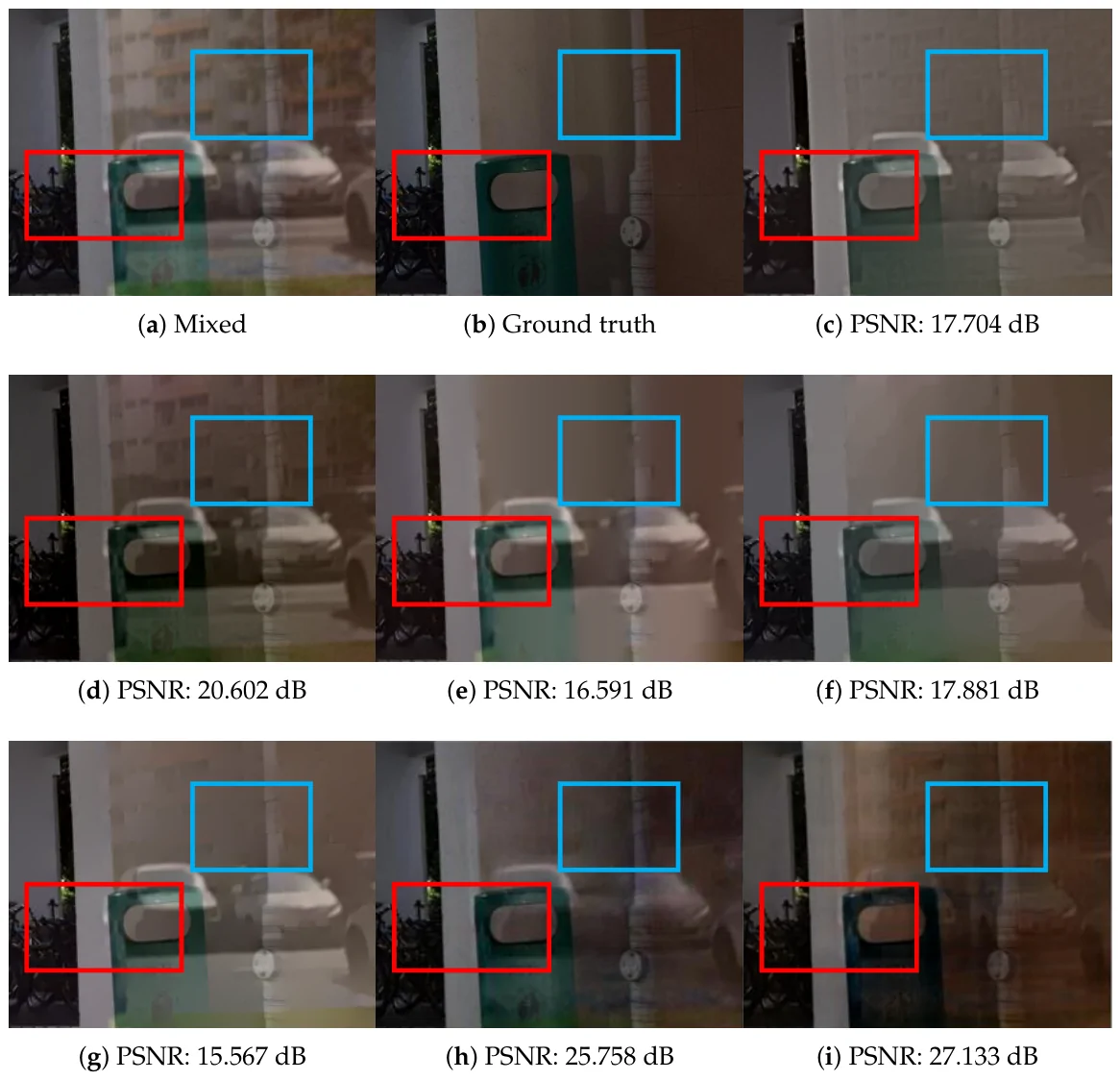

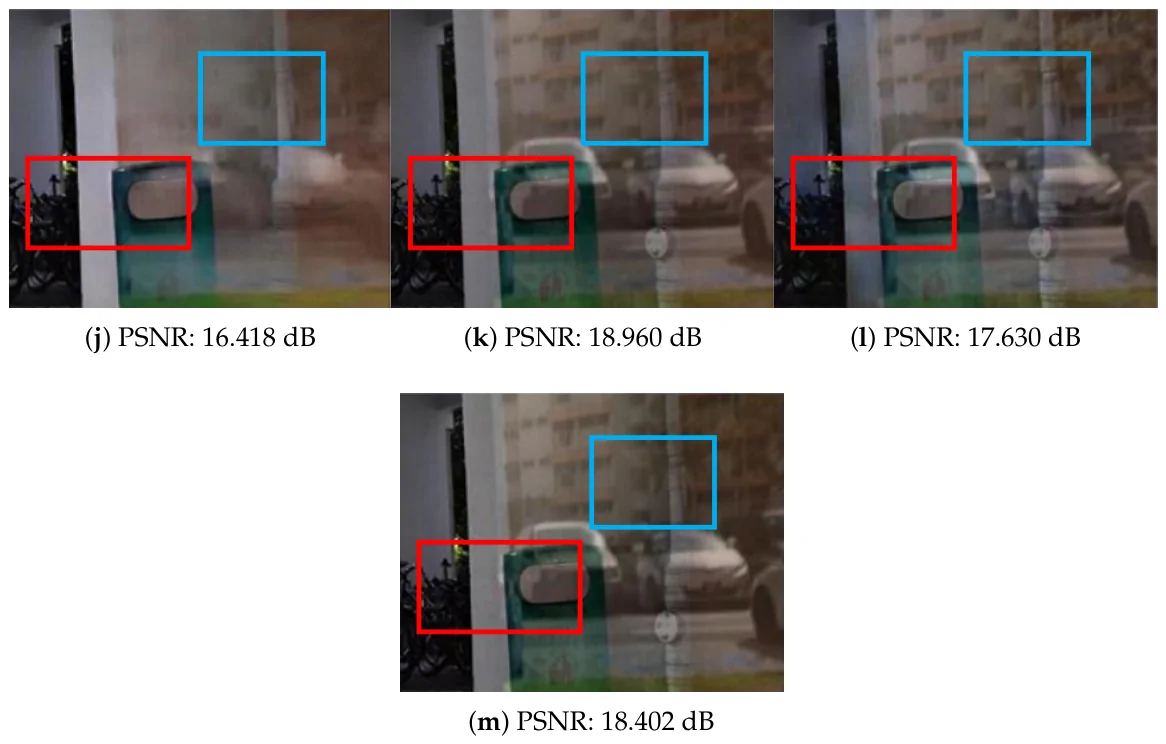
Qualitative comparison of proposed method with competitive non-learning and deep learning methods on outdoor dataset: (**a**) Mixture image, (**b**) Ground truth, (**c**) Proposed, (**d**) LB14 [13], (**e**) WS16 [29], (**f**) NR17 [25], (**g**) YW19 [7], (**h**) CoRRN [44], (**i**) ZN18 [36], (**j**) YD18 [37], (**k**) ERRNet [45], (**l**) SIRRBL [46], (**m**) IBCLN [47]. Red and blue boxes indicate the two regions of interest (ROIs) selected for magnified comparison across all methods.

**Figure 13:**
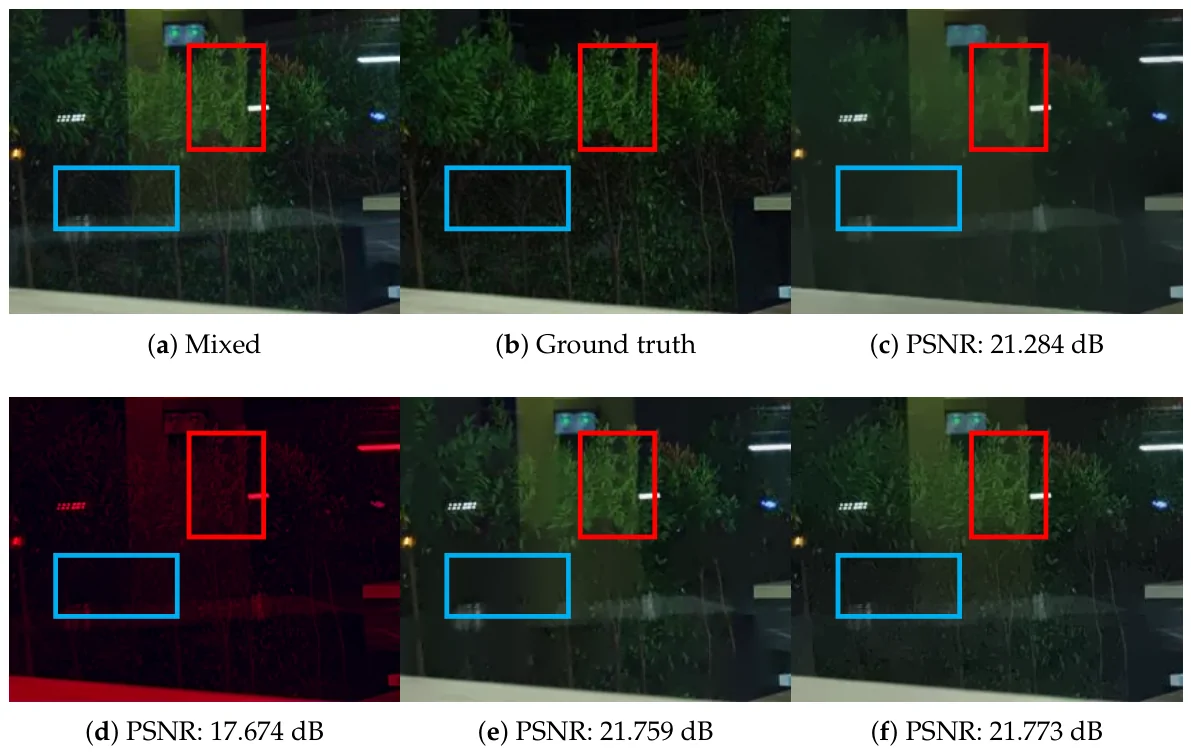

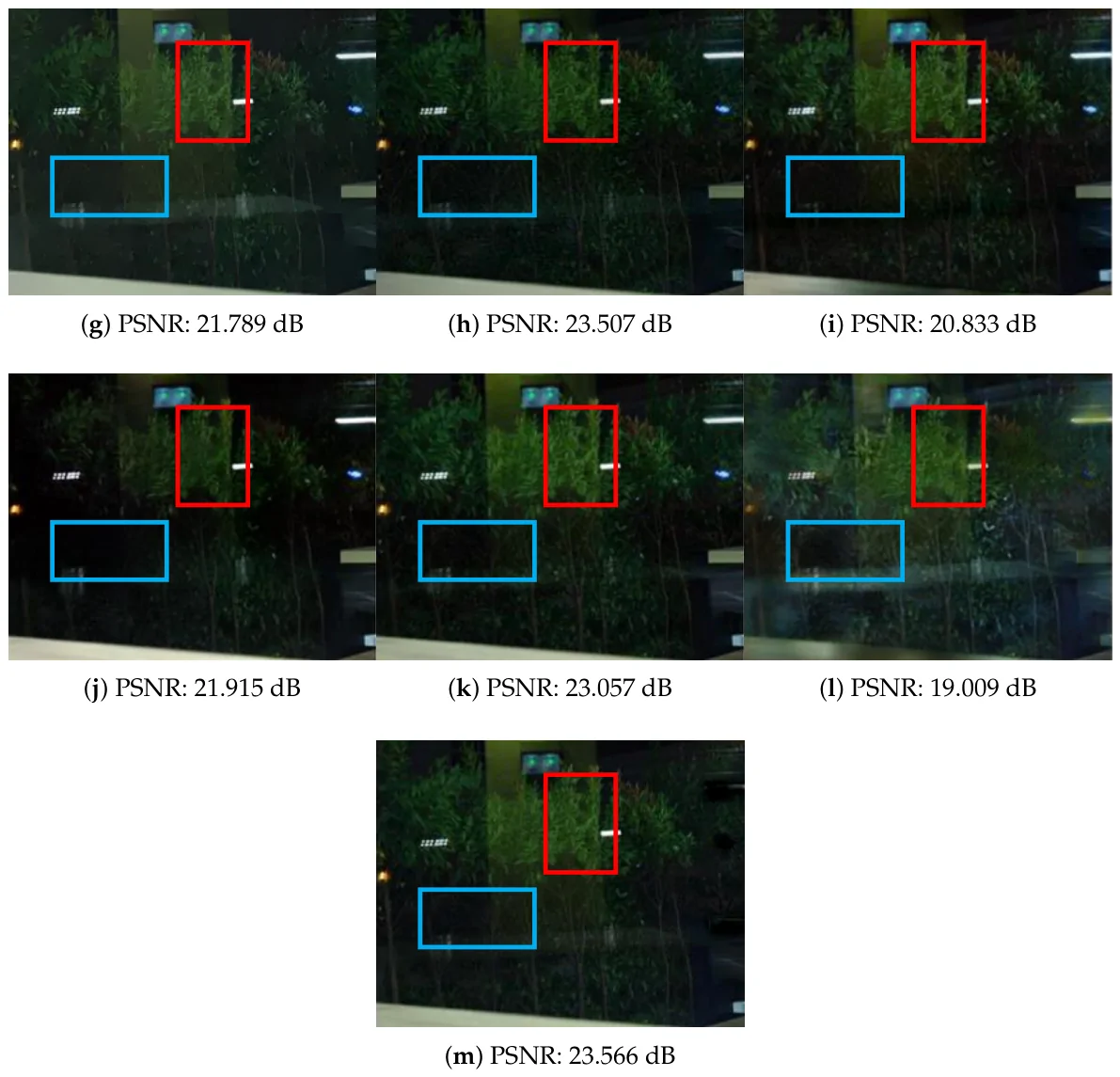
Qualitative comparison of proposed method with competitive non-learning and deep learning methods on outdoor dataset: (**a**) Mixture image, (**b**) Ground truth, (**c**) Proposed, (**d**) LB14 [13], (**e**) WS16 [29], (**f**) NR17 [25], (**g**) YW19 [7], (**h**) CoRRN [44], (**i**) ZN18 [36], (**j**) YD18 [37], (**k**) ERRNet [45], (**l**) SIRRBL [46], (**m**) IBCLN [47]. Red and blue boxes indicate the two regions of interest (ROIs) selected for magnified comparison across all methods.

**Figure 14:**
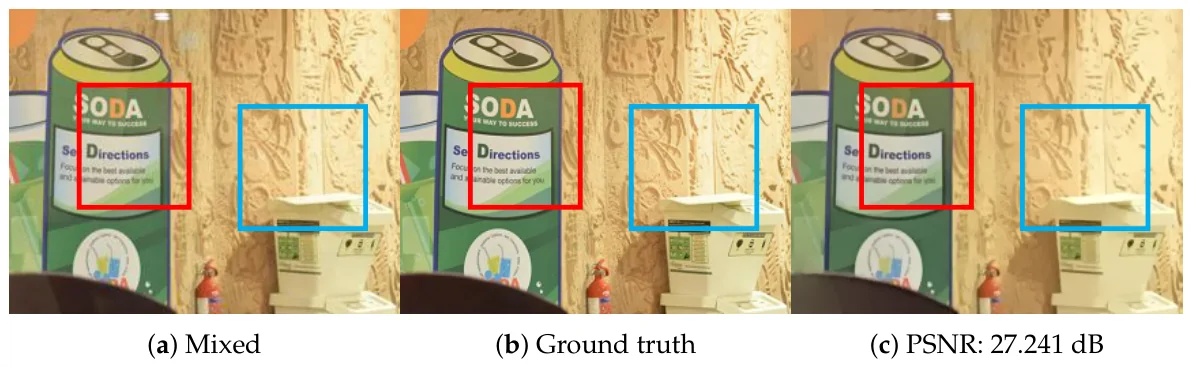

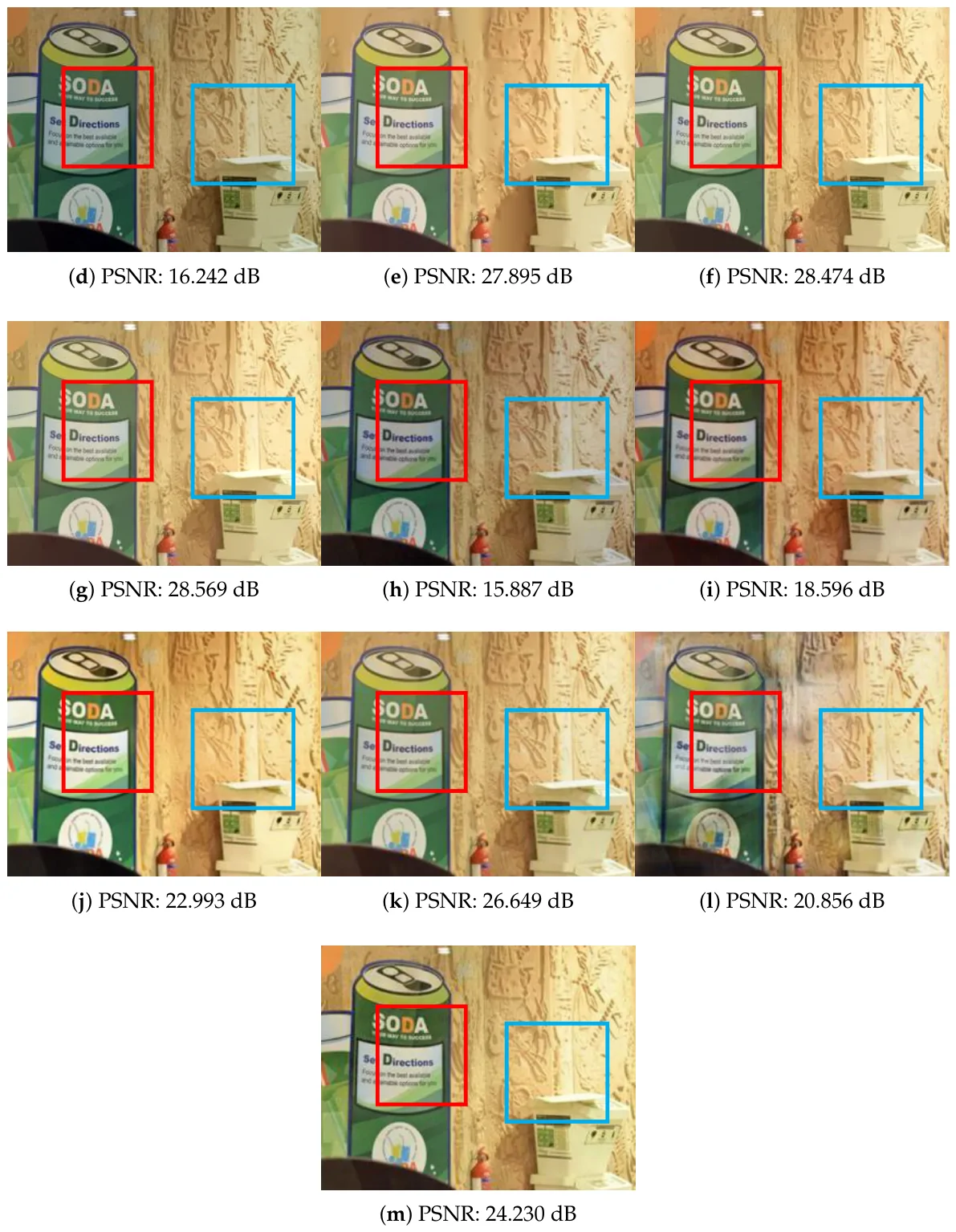
Qualitative comparison of proposed method with competitive non-learning and deep learning methods on outdoor dataset: (**a**) Mixture image, (**b**) Ground truth, (**c**) Proposed, (**d**) LB14 [13], (**e**) WS16 [29], (**f**) NR17 [25], (**g**) YW19 [7], (**h**) CoRRN [44], (**i**) ZN18 [36], (**j**) YD18 [37], (**k**) ERRNet [45], (**l**) SIRRBL [46], (**m**) IBCLN [47]. Red and blue boxes indicate the two regions of interest (ROIs) selected for magnified comparison across all methods.

Figure 11, Figure 12, Figure 13 and Figure 14 further demonstrate results from evaluating performance on outdoor scenes containing vegetation, urban structures, and varying illumination conditions, including grass and building scenes, street-level views, low-light vegetation, and mixed indoor–outdoor setups. Figure 11 illustrates a complex outdoor property setting used to test the methods for texture preservation, edge sharpness, and color integrity. When analyzing the foreground lawn, the conventional method WS16 [29] replaces the fine grass texture with a washed-out yellowish-green blur; the deep learning model SIRRBL [46] introduces an unnatural blue artifact layer; and methods NR17 [25], YW19 [7], ZN18 [36], and IBCLN [47] leave prominent vertical reflection bands; meanwhile, the proposed method substantially suppresses these artifacts to retain a sharp, high-contrast texture. For the white car parked on the right, the baseline method LB14 [13] introduces a dark contrast shift, while the methods CoRRN [44] and SIRRBL [46] generate localized blurring and halos, whereas the proposed method reconstructs the vehicle with sharp edges and a largely natural white balance. Evaluating the results shown in Figure 12, methods LB14 [13], CoRRN [44], and IBCLN [47] cause muddy underexposure; the method ZN18 [36] injects a strong global orange color cast; and methods WS16 [29] and YD18 [37] introduce structural blurring. In contrast, the proposed method largely preserves the sharp geometric transitions with accurate color saturation. Finally, across the global background wall, methods LB14 [13] and CoRRN [44] suffer from severe contrast collapse, the method ZN18 [36] causes extreme sepia discoloration, the method SIRRBL [46] creates a pixelated glow, and methods WS16 [29] and YD18 [37] produce a cloudy, compressed haze. Although the proposed method does not completely remove the car reflection in this specific dataset, it maintains the correct global color temperature of the scene. Figure 13 shows the results from testing reflection removal and texture retention over natural dense greenery and bushes. Focusing on the upper-center vertical pillar reflection where a bright rectangular block heavily obscures the greenery in the mixed input, conventional baselines WS16 [29], NR17 [25], and YW19 [7] and deep learning models ERRNet [45] and IBCLN [47] largely retain the sharp contours of the artifact. Meanwhile, the method LB14 [13] introduces a severe dark red color cast that degrades most structural content. Only the deep learning model CoRRN [44] removes the vertical block to recover a uniform dark background in close alignment with the ground truth. The proposed method, although it diffuses the sharp boundaries of the rectangular pillar, retains the underlying organic textures notably better than these conventional approaches; it shows a shortcoming in contrast loss, leaving a hazy overlay. For the sharp horizontal glare band cutting across the lower half of the foliage, baselines WS16 [29], NR17 [25], and YW19 [7] leave the artifact largely untouched, failing to separate the intersecting layers. Deep learning baselines CoRRN [44] and IBCLN [47] also struggle to decouple this region, keeping the sharp streak contours visible. Meanwhile, methods ZN18 [36] and YD18 [37] introduce severe underexposure and dark color shifts that obscure the lower vegetation details. Additionally, the deep learning framework SIRRBL [46] introduces severe noise distortion, scattering a hazy, blue-white speckled glare over the lower half of the frame. In contrast, both the deep learning model ERRNet [45] and the proposed method remove this prominent horizontal streak comparatively well and without generating substantial secondary noise artifacts. While the proposed approach experiences minor contrast loss, it clears the lower field of view to expose the structural distribution of the foliage, broadly matching the overall spatial layout of the ground truth. Figure 14 shows the results from examining color saturation, text clarity, and multi-layered glare suppression across an indoor poster and a textured wall display. Looking at the green soda can poster on the left half of the frame, the deep learning method CoRRN [44] undergoes severe contrast and color collapse that turns the vivid green into a desaturated gray, the method LB14 [13] introduces an unnatural dark exposure shift, and the method SIRRBL [46] allows a hazy white light leakage to bleed over and blur the structural border. The proposed method avoids most of these distortions, maintaining strong color harmony, contrast, and textual detail on the poster. On the right side of the frame, where the textured wall and electronic device are heavily occluded by widespread background glare in the mixed input, conventional baselines NR17 [25] and YW19 [7] and deep learning models YD18 [37], ERRNet [45], and IBCLN [47] fail to suppress this global glare, leaving the washed-out appearance largely intact. Most noticeably, the deep learning framework SIRRBL [46] introduces a smoky, cloud-like haze on the upper-right portion of the textured wall. The proposed method compares favorably against these approaches by suppressing the reflection layers with minimal added haze or noise, restoring the organic textures of the wall and the geometric boundaries of the device closely in line with the ground truth. While the proposed framework exhibits minor color-tuning adjustments to compensate for intricate ambient lighting paths, it largely preserves native color fidelity—such as the vibrant green lawn and dark night tones—producing clean, visually plausible background restorations.

**Figure 15:**
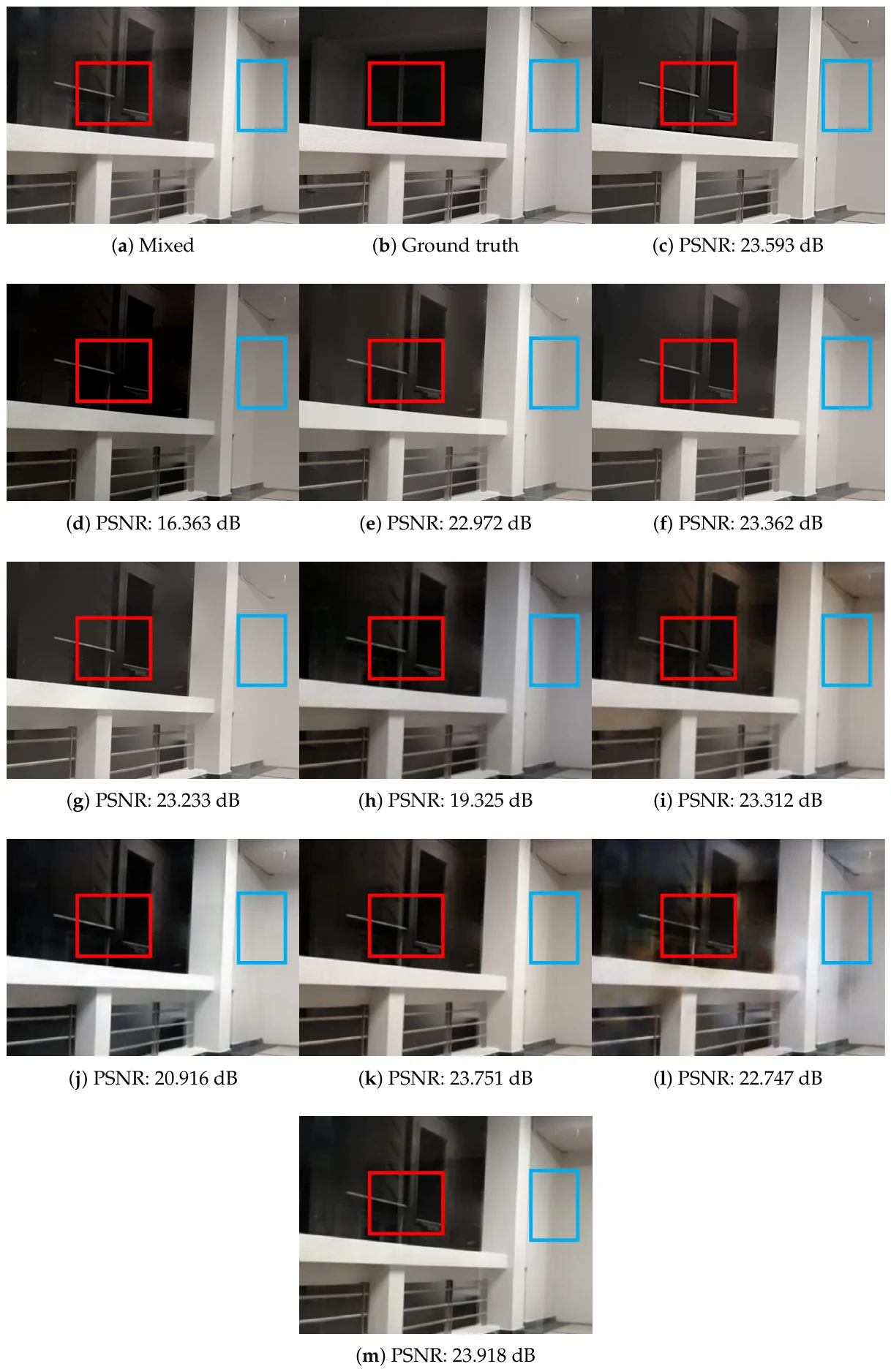
Qualitative comparison of proposed method with competitive non-learning and deep learning methods on In-The-Wild dataset: (**a**) Mixture image, (**b**) Ground truth, (**c**) Proposed, (**d**) LB14 [13], (**e**) WS16 [29], (**f**) NR17 [25], (**g**) YW19 [7], (**h**) CoRRN [44], (**i**) ZN18 [36], (**j**) YD18 [37], (**k**) ERRNet [45], (**l**) SIRRBL [46], (**m**) IBCLN [47]. Red and blue boxes indicate the two regions of interest (ROIs) selected for magnified comparison across all methods.

**Figure 16:**
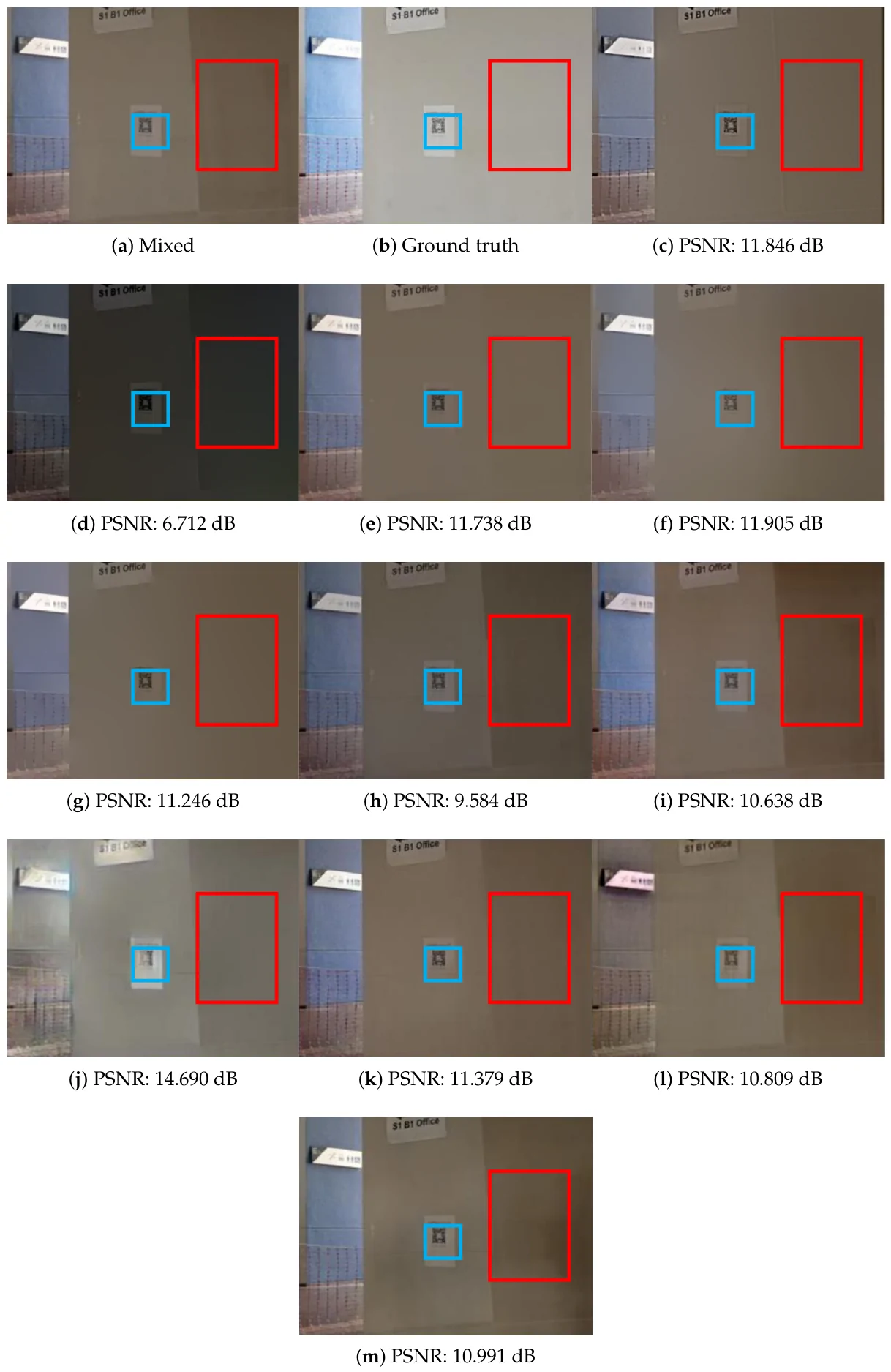
Qualitative comparison of proposed method with competitive non-learning and deep learning methods on In-The-Wild dataset: (**a**) Mixture image, (**b**) Ground truth, (**c**) Proposed, (**d**) LB14 [13], (**e**) WS16 [29], (**f**) NR17 [25], (**g**) YW19 [7], (**h**) CoRRN [44], (**i**) ZN18 [36], (**j**) YD18 [37], (**k**) ERRNet [45], (**l**) SIRRBL [46], (**m**) IBCLN [47]. Red and blue boxes indicate the two regions of interest (ROIs) selected for magnified comparison across all methods.

Finally, Figure 15 and Figure 16 illustrate the results from assessing generalization capability on the In-The-Wild dataset, which contains real-world images captured under unconstrained imaging conditions where reflection intensity, lighting, and scene composition vary significantly. Figure 15 illustrates the results from evaluating a structure featuring dark window glass panes beside a white vertical wall panel; the models are tested on their capability to separate strong reflections and prevent localized color degradation. In the dark glass window area on the left half of the frame where a soft, pale vertical reflection mask stretches across the surface in the mixed input, conventional baselines NR17 [25] and YW19 [7] and deep learning models ZN18 [36], ERRNet [45], and IBCLN [47] leave these reflection overlays largely untouched. Furthermore, methods LB14 [13] and CoRRN [44] overcompensate by severely underexposing the entire image, turning the crisp white architecture into a dark, muddy gray. Meanwhile, the method SIRRBL [46] introduces a smoky white glare artifact over the glass. In contrast, the proposed method attenuates the soft reflection veil on the left window pane, recovering a cleaner dark glass surface. However, this does show a limitation in fully decoupling high-intensity structures, as the bright rectangular reflection frame on the glass surface to the right remains partially visible compared to the clean black void of the ground truth. Along the rightmost white vertical wall panel and corner, many baseline models struggle significantly: methods LB14 [13] and CoRRN [44] introduce severe vignetting and edge shadows that turn the wall dark gray, while methods WS16 [29], ZN18 [36], and ERRNet [45] introduce a muddy, warm yellowish color tint. Most notably, the deep learning framework SIRRBL [46] introduces substantial artifacts, creating a heavily distorted gray–blue smudge that degrades the wall’s texture and structure. The baseline method YD18 [37] is largely successful at removing the window glass reflections; however, its aggressive suppression erases the sharp corner edges and architectural lines where the window frames meet the wall. The proposed method demonstrates strong color fidelity, a largely neutral white across the walls, and sharp geometric boundaries along the railing. While the proposed approach leaves a minor, faint remnant of the intense rectangular reflection frame on the right glass pane, it avoids the substantial structural loss seen in YD18 [37] and the global color distortion seen in ERRNet [45], striking a balanced trade-off between reflection suppression and scene fidelity.

Figure 16 focuses on a wall partition featuring a broad reflection mask and a small central sticker, which were used to evaluate non-uniform layer suppression and local contrast preservation. Looking at the large vertical reflection zone on the right half of the partition where a distinct dark brown reflection mask heavily covers the wall in the mixed image, competing baselines struggle significantly: methods CoRRN [44], ZN18 [36], ERRNet [45], SIRRBL [46], and IBCLN [47] leave the dark brown shadow layer largely intact; the method LB14 [13] collapses into severe global underexposure; and the method YD18 [37] fails to reconstruct a consistent surface texture. The proposed method suppresses most of this broad reflection layer, recovering a largely smooth, uniform wall surface close to the clean background profile established by the ground truth. Centering on the small central sticker and its surrounding local contrast, the baseline method NR17 [25] severely washes out the sticker’s local contrast, making it fade into the background. Most notably, the deep learning framework YD18 [37] introduces a substantial localized artifact, creating an unnatural, bright white boxy halo directly around the sticker that obscures much of the surrounding wall detail. By avoiding these artifacts, the proposed method closely preserves the boundary, contrast, and visibility of the sticker element, demonstrating strong structural fidelity and texture preservation.

## 4. Discussion and Limitations

### 4.1. Theoretical Contribution and Motivation

Beyond the empirical performance reported below, the core theoretical contribution of this work lies in the combination and adaptation of established components from prior structure–texture-based reflection removal literature, which differs from existing full-pipeline approaches. The closest prior work following a similar structure–texture decomposition strategy is Jiang et al. [3], which also builds on the total variation decomposition of Xu et al. [2] and the thresholding scheme of Yang et al. [7]. However, Jiang et al. apply thresholding to both the structure and texture layers independently, whereas the proposed method computes a single mask from the structure layer and applies it to the uniform smoothing output, since layer separation concentrates most of the background content in the structure layer, making it a more reliable source for mask estimation. Jiang et al. further rely on a coefficient-weighted DCT-domain de-blocking step and recombine the layers through fixed linear fusion, while the proposed method instead uses a dedicated scene-detail mask refined through soft matting and recombines the layers through DCP-weighted scaling.

The hard thresholding stage also extends the fixed global threshold *h* used by Yang et al. [7], by reformulating single-image reflection thresholding as a spatially varying, hierarchical decision process. By first establishing a per-region brightness classification via Otsu’s method and then deriving distinct local statistics-based thresholds for bright and dark regions, the proposed hierarchical framework explicitly accommodates the co-occurrence of high- and low-illumination content within a single scene, a condition under which a single global threshold necessarily over- or under-suppresses one region. This reformulation extends threshold-based reflection suppression beyond the largely uniform-illumination assumptions implicit in prior structure–texture pipelines.

This work is further motivated by the observation that the reflection removal literature has increasingly concentrated around deep learning solutions requiring large-scale paired training data, GPU acceleration, and substantial model capacity, often at the cost of generalization to unseen reflection types and deployment on training- or GPU-constrained platforms. The traditional, optimization-based route adopted here is motivated by the premise that competitive perceptual-quality reflection suppression remains achievable without a training pipeline, by combining classical structure–texture decomposition, adaptive hierarchical thresholding, and perceptually-guided recombination, offering a deployment path for settings where paired training data, GPU access, or model retraining are impractical.

### 4.2. Overall Performance

The experimental results presented in the previous sections demonstrate that the proposed reflection removal framework effectively suppresses reflection components while preserving perceptual image quality across diverse imaging conditions. The quantitative evaluations on the indoor, outdoor, and In-The-Wild datasets, assessed using both full-reference and no-reference metrics, reveal a consistent performance profile that reflects the fundamental design philosophy of our approach: prioritizing perceptual naturalness and artifact suppression over aggressive pixel-wise reconstruction.

On the indoor dataset, our method achieves competitive reconstruction fidelity (PSNR: 21.448, SSIM: 0.836) while securing top-three rankings across all no-reference perceptual metrics (BRISQUE: 27.584, NIQE: 3.655, PIQE: 55.019), outperforming all deep learning competitors on each. This trend extends to the outdoor dataset, where our method leads the deep learning methods on BRISQUE (28.478) and PIQE (59.719), while also surpassing methods such as YD18 [37] and SIRRBL [46] in PSNR. Qualitative results corroborate these findings, with our method consistently producing visually coherent outputs largely free from the over-smoothing, halos, and ghosting artifacts that affect several competing approaches.

### 4.3. Generalization and Robustness

A notable strength of the proposed method is its generalization capability across domains. The parameter sensitivity analyses for aperture variation (F11 vs. F32) and glass thickness variation (T3 vs. T10) confirm that our approach remains stable under changing acquisition conditions, with minimal performance fluctuation despite the varying reflection sharpness or glass thickness. This robustness is particularly evident in this experiment, where our method maintains near-identical performance when moving from the smallest spatial shift (T3) to the largest (T10), with fluctuation smallest than that observed for most competing deep learning methods. The In-The-Wild results further underscore this generalization, as our method produces visually plausible restorations under unconstrained imaging conditions where reflection intensity, lighting, and scene composition vary significantly. These findings indicate that our approach does not overfit to the specific acquisition parameters of the controlled benchmark, supporting its applicability in less constrained environments.

### 4.4. Perception–Distortion Trade-Off

While our method performs strongly on perceptual quality metrics, the quantitative evaluations also reveal a systematic trade-off that warrants discussion. On the more challenging In-The-Wild dataset, our method exhibits a drop in local structural metrics compared to other deep learning approaches, obtaining the lowest Local_NCC (0.402) and the highest (worst) LMSE (0.027) among the deep learning methods evaluated. This observation is consistent with the well-established perception–distortion trade-off in image restoration literature [48]. Methods that achieve stronger fidelity metrics—such as IBCLN with a higher PSNR (15.841) or ZN18 and IBCLN with higher SSIM scores (0.609 and 0.619, respectively)—may do so in part by favoring smoother reconstructions that increase structural similarity at some cost to high-frequency texture preservation; although, we note this attribution is inferred from the observed metric pattern rather than directly verified against each method’s implementation. Our method instead prioritizes preserving natural textural variation, which is consistent with both our lower local correlation scores and our stronger performance in BRISQUE (34.189, best among all evaluated methods) and PIQE (59.517, best among the deep learning methods) among the no-reference perceptual metrics, though this comes with a corresponding rise in NIQE on both the outdoor and In-The-Wild datasets (discussed in the Section 4.5 section below).

### 4.5. Limitations

Despite the promising results, four core limitations of the proposed method should be acknowledged.

- **Poor pixel-wise fidelity under unconstrained conditions.** While the method achieves competitive PSNR and SSIM on the controlled indoor and outdoor datasets, its full-reference fidelity degrades more substantially on the In-The-Wild dataset, with the lowest Local_NCC (0.402) and highest (worst) LMSE (0.027) among the deep learning methods evaluated. This stems directly from the method’s design philosophy: the hierarchical thresholding, DCT-based masking, and DCP-weighted recombination stages are optimized to suppress reflection artifacts and preserve perceptual naturalness, but no stage explicitly minimizes pixel-wise reconstruction error against the ground truth. Under the wider variability of unconstrained scenes, this absence of an explicit fidelity constraint allows local structural deviations to accumulate more than it does under the narrower conditions of the controlled datasets.
- **Single-dataset benchmark reliance.** All quantitative results, including the In-The-Wild evaluation, are drawn from a single benchmark source with pre-defined acquisition parameters (aperture, glass thickness). While this enables controlled, systematic comparison across methods, it also means the reported generalization has not been verified against independently collected real-world reflection datasets, which may exhibit reflection characteristics (e.g., surface curvature, multi-layer reflections, mixed indoor–outdoor lighting) not represented in this benchmark’s construction.
- **No lightweight computational quantification.** Although the proposed method requires no GPU acceleration, large-scale training data, or specialized hardware, unlike the deep learning baselines, its measured runtime (33.658 s, on an 11th Gen Intel Core i5-1135G7, CPU-only) is substantially higher than nearly all deep learning baselines evaluated (as low as 0.013 s, GPU-based, literature-reported) and higher than most of the traditional baselines. This cost is attributable primarily to the L0 gradient-based uniform smoothing stage, which iteratively solves a large sparse linear system per image via ADAM-based optimization; while this stage contributes materially to the method’s perceptual-quality advantage, it is also the primary obstacle to genuine lightweight or real-time deployment. Therefore, the method’s hardware-independence should not be conflated with computational efficiency.
- **No downstream task validation.** The evaluation is restricted to standard full-reference and no-reference image-quality metrics. No experiments assess whether the proposed reflection removal improves performance on downstream computer vision tasks, such as object detection, semantic segmentation, or visual SLAM, that would be practically affected by residual reflection artifacts or by the textural changes introduced by the DCP-weighted recombination. Because such tasks are sensitive to failure modes—e.g., false edges from residual reflection or localized texture shifts—that are not directly captured by BRISQUE, NIQE, PIQE, or pixel-wise metrics, the practical utility of the method’s outputs for downstream applications remains unverified.

Separately, the NIQE metric appears less favorable to our approach on both the outdoor and In-The-Wild datasets, recording the highest (least favorable) value among deep learning competitors despite securing the top results in BRISQUE and PIQE among that same group on both datasets. This discrepancy is likely attributable to our deliberate preservation of high-frequency textural variation, which deviates from the rigid Natural Scene Statistics (NSS) models expected by NIQE, particularly in uniform regions such as skies and vegetation. While BRISQUE and PIQE are trained on human-rated distortions and therefore correlate more closely with subjective quality, the elevated NIQE scores indicate that our method may not satisfy all statistical assumptions of conventional no-reference quality assessment frameworks.

### 4.6. Future Directions

Building on the limitations identified above, future work is organized into three phases, each targeting a specific technical bottleneck.

- **Phase 1 (near-term): Validating current claims.** The most immediate priority is conducting a subjective user study in which volunteers rate the outputs of all compared methods on a five-point scale, aggregated into a Mean Opinion Score (MOS) following an ITU-R BT.500-style protocol, to statistically substantiate the naturalness advantage suggested by our no-reference metrics. The core bottleneck here is participant recruitment and rating-protocol design rather than a technical limitation of the method itself.
- **Phase 2 (mid-term): Closing the local-fidelity and efficiency gaps.** To address the local structural fidelity gap identified in the Limitations section, we plan to develop an adaptive edge-aware refinement module that activates specifically in high-entropy, unstructured regions. The core technical bottleneck is designing a local-consistency term that improves patch-level correlation without reintroducing suppressed reflection content or oversmoothing genuine detail, since these two failure modes pull the refinement in opposite directions. In parallel, to address the computational-cost limitation, we plan to investigate lower-cost alternatives to the L0 gradient-based uniform smoothing stage, with the bottleneck being whether a cheaper approximation can retain the perceptual-quality gains currently attributable to this stage.
- **Phase 3 (long-term): Reducing dataset dependency and broadening validation.** We plan to explore hybrid perceptual losses that incorporate relaxed NSS constraints to reduce the NIQE penalty without reintroducing over-smoothing; the bottleneck is balancing relaxed NSS regularization against the risk of reintroducing the artifacts the method currently avoids. We additionally plan to investigate unsupervised or self-supervised strategies to reduce reliance on pre-defined dataset configurations, and to validate the method against additional real-world datasets to address the single-dataset limitation. Finally, we plan to evaluate the integration of the proposed framework with downstream tasks such as object detection and semantic segmentation, directly addressing the lack of downstream validation identified above; the bottleneck here is establishing task-specific metrics sensitive to the residual artifact types this method’s evaluation has not yet captured.

## Figures and Tables

**Table 1:** The ablation study of the proposed method on the ‘In-The-Wild’ dataset. ↑ and ↓ denote that higher and lower values are better, respectively. The color transitions show the fluctuating metric values across the pipeline stages.

Metric	Uniform Smoothing	Hard Thresholding	Structure–Texture Decomposition	DCT-Based Mask Estimation	Soft Matting	Final Reconstruction
↑ PSNR	15.275	15.201	15.176	15.101	15.132	15.745
↓ MSE	0.043	0.044	0.044	0.044	0.044	0.042
↑ SSIM	0.541	0.535	0.532	0.512	0.518	0.569
↑ Global	0.756	0.731	0.729	0.717	0.719	0.762
↑ Local	0.392	0.346	0.345	0.316	0.327	0.402
↓ LMSE	0.037	0.038	0.039	0.042	0.041	0.027
↑ SI	0.507	0.489	0.488	0.485	0.492	0.559
↓ BRISQUE	33.454	38.763	31.806	36.171	34.936	34.189
↓ NIQE	5.192	6.513	5.289	5.862	5.881	5.789
↓ PIQE	50.570	55.844	49.301	49.590	56.068	59.517

Color coding: Dark Blue = best, Lightest Blue = worst.

**Table 2:** The average error metric values of non-learning methods compared to the proposed method on the indoor dataset. ↑ and ↓ denote the higher and lower values are better, respectively.

	Proposed	LB14 [13]	WS16 [29]	NR17 [25]	YW19 [7]
↑ PSNR	21.448	12.046	15.218	15.544	16.395
↓ MSE	0.008	0.081	0.065	0.062	0.060
↑ SSIM	0.836	0.450	0.515	0.526	0.529
↑ Global NCC	0.958	0.542	0.545	0.544	0.549
↑ Local NCC	0.728	0.425	0.352	0.389	0.382
↓ LMSE	0.008	0.080	0.068	0.068	0.067
↑ SI	0.838	0.483	0.497	0.523	0.518
↓ BRISQUE	27.584	26.196	41.614	39.269	37.437
↓ NIQE	3.655	3.622	4.581	5.167	4.319
↓ PIQE	55.019	36.188	57.021	56.639	51.669

Color coding: Green = best, Orange = second-best.

**Table 3:** The average error metric values of deep learning methods compared to the proposed method on the indoor dataset. ↑ and ↓ denote the higher and lower values are better, respectively.

	Proposed	CoRRN [44]	ZN18 [36]	YD18 [37]	ERRNet [45]	SIRRBL [46]	IBCLN [47]
↑ PSNR	21.448	19.170	22.560	23.112	20.414	22.347	18.562
↓ MSE	0.008	0.018	0.006	0.006	0.010	0.006	0.015
↑ SSIM	0.836	0.706	0.843	0.856	0.795	0.828	0.714
↑ Global NCC	0.958	0.943	0.950	0.959	0.927	0.944	0.861
↑ Local NCC	0.728	0.736	0.765	0.768	0.662	0.745	0.639
↓ LMSE	0.008	0.011	0.007	0.007	0.014	0.010	0.020
↑ SI	0.838	0.668	0.844	0.846	0.795	0.825	0.751
↓ BRISQUE	27.584	38.262	39.154	36.931	38.313	38.098	39.657
↓ NIQE	3.655	3.831	4.078	3.767	4.122	4.090	4.119
↓ PIQE	55.019	66.923	63.498	63.899	66.189	65.847	64.936

Color coding: Green = best, Orange = second-best.

**Table 4:** The average error metric values of non-learning methods compared to the proposed method on the outdoor dataset. ↑ and ↓ denote the higher and lower values are better, respectively.

	Proposed	LB14 [13]	WS16 [29]	NR17 [25]	YW19 [7]
↑ PSNR	21.758	20.655	22.874	23.746	21.289
↓ MSE	0.009	0.013	0.009	0.007	0.012
↑ SSIM	0.859	0.778	0.885	0.894	0.859
↑ Global NCC	0.884	0.888	0.896	0.923	0.890
↑ Local NCC	0.670	0.690	0.684	0.673	0.664
↓ LMSE	0.022	0.032	0.018	0.018	0.018
↑ SI	0.901	0.826	0.914	0.917	0.916
↓ BRISQUE	28.478	26.364	43.242	41.618	41.400
↓ NIQE	4.833	4.554	6.190	7.218	6.173
↓ PIQE	59.719	52.280	63.405	63.194	61.093

Color coding: Green = best, Orange = second-best.

**Table 5:** The average error metric values of deep learning methods compared to the proposed method on the outdoor dataset. ↑ and ↓ denote the higher and lower values are better, respectively.

	Proposed	CoRRN [44]	ZN18 [36]	YD18 [37]	ERRNet [45]	SIRRBL [46]	IBCLN [47]
↑ PSNR	21.758	24.327	23.356	20.938	24.968	20.909	23.107
↓ MSE	0.009	0.006	0.007	0.011	0.005	0.010	0.007
↑ SSIM	0.859	0.886	0.862	0.841	0.904	0.799	0.887
↑ Global NCC	0.884	0.942	0.927	0.934	0.928	0.861	0.910
↑ Local NCC	0.670	0.680	0.660	0.666	0.685	0.610	0.653
↓ LMSE	0.022	0.012	0.015	0.014	0.016	0.021	0.017
↑ SI	0.901	0.886	0.879	0.868	0.914	0.821	0.894
↓ BRISQUE	28.478	41.099	41.595	40.438	42.082	41.470	42.109
↓ NIQE	4.833	4.451	4.535	4.525	4.564	4.552	4.487
↓ PIQE	59.719	67.062	71.792	72.656	70.172	69.468	69.291

Color coding: Green = best, Orange = second-best.

**Table 6:** The average error metric values of non-learning methods compared to the proposed method on the In-The-Wild dataset. ↑ and ↓ denote the higher and lower values are better, respectively.

	Proposed	LB14 [13]	WS16 [29]	NR17 [25]	YW19 [7]
↑ PSNR	15.745	12.617	14.546	15.834	15.236
↓ MSE	0.042	0.097	0.050	0.041	0.043
↑ SSIM	0.569	0.480	0.560	0.585	0.574
↑ Global NCC	0.762	0.781	0.712	0.789	0.766
↑ Local NCC	0.402	0.439	0.396	0.430	0.408
↓ LMSE	0.027	0.032	0.027	0.024	0.026
↑ SI	0.559	0.511	0.554	0.563	0.550
↓ BRISQUE	34.189	34.837	49.585	41.606	45.411
↓ NIQE	5.789	4.959	6.150	6.641	6.913
↓ PIQE	59.517	41.129	72.587	62.871	68.748

Color coding: Green = best, Orange = second-best.

**Table 7:** The average error metric values of deep learning methods compared to the proposed method on the In-The-Wild dataset. ↑ and ↓ denote the higher and lower values are better, respectively.

	Proposed	CoRRN [44]	ZN18 [36]	YD18 [37]	ERRNet [45]	SIRRBL [46]	IBCLN [47]
↑ PSNR	15.745	13.830	15.146	16.134	15.190	15.227	15.841
↓ MSE	0.042	0.059	0.048	0.035	0.049	0.046	0.045
↑ SSIM	0.569	0.599	0.609	0.588	0.588	0.556	0.619
↑ Global NCC	0.762	0.872	0.865	0.791	0.882	0.850	0.855
↑ Local NCC	0.402	0.582	0.569	0.505	0.590	0.520	0.578
↓ LMSE	0.027	0.020	0.018	0.023	0.019	0.020	0.019
↑ SI	0.559	0.614	0.685	0.573	0.660	0.526	0.680
↓ BRISQUE	34.189	46.891	45.498	43.364	46.040	47.108	46.905
↓ NIQE	5.789	4.557	4.443	4.383	4.485	4.296	4.277
↓ PIQE	59.517	75.554	78.048	76.919	77.505	76.571	76.192

Color coding: Green = best, Orange = second-best.

**Table 8:** Evaluation of proposed method compared to the non-learning methods using indoor data set with F-variance. ↑ and ↓ denote the higher and lower values are better, respectively.

Fvar	Proposed	LB14 [13]	WS16 [29]	NR17 [25]	YW19 [7]
↑ PSNR					
(F11)	21.234	11.721	15.081	15.386	16.603
(F32)	21.487	12.051	15.254	15.610	16.358
↓ MSE					
(F11)	0.008	0.084	0.065	0.063	0.060
(F32)	0.008	0.081	0.065	0.062	0.061
↑ SSIM					
(F11)	0.837	0.445	0.514	0.527	0.533
(F32)	0.838	0.452	0.516	0.530	0.530
↑ Global NCC					
(F11)	0.960	0.545	0.549	0.546	0.554
(F32)	0.958	0.543	0.545	0.545	0.549
↑ Local NCC					
(F11)	0.740	0.431	0.359	0.385	0.381
(F32)	0.724	0.427	0.350	0.395	0.387
↓ LMSE					
(F11)	0.008	0.077	0.066	0.065	0.065
(F32)	0.008	0.081	0.069	0.068	0.068
↑ SI					
(F11)	0.846	0.489	0.495	0.527	0.524
(F32)	0.841	0.482	0.498	0.527	0.523
↓ BRISQUE					
(F11)	27.455	23.883	43.435	40.969	40.695
(F32)	27.335	25.945	40.766	38.230	35.396
↓ NIQE					
(F11)	3.840	3.670	4.816	5.386	4.604
(F32)	3.473	3.501	4.435	5.023	4.188
↓ PIQE					
(F11)	55.338	40.483	60.154	60.002	55.108
(F32)	54.818	33.844	55.248	54.802	49.814

Color coding: Green = best, Orange = second-best.

**Table 9:** Evaluation of proposed method compared to the deep learning methods using indoor data set with F-variance. ↑ and ↓ denote the higher and lower values are better, respectively.

Fvar	Proposed	CoRRN [44]	ZN18 [36]	YD18 [37]	ERRNet [45]	SIRRBL [46]	IBCLN [47]
↑ PSNR							
(F11)	21.234	19.281	22.777	22.985	20.253	22.411	18.506
(F32)	21.487	18.905	22.392	22.995	20.424	22.181	18.545
↓ MSE							
(F11)	0.008	0.018	0.006	0.006	0.010	0.006	0.015
(F32)	0.008	0.019	0.006	0.006	0.010	0.007	0.015
↑ SSIM							
(F11)	0.837	0.719	0.851	0.857	0.797	0.836	0.720
(F32)	0.838	0.699	0.842	0.854	0.795	0.825	0.715
↑ Global NCC							
(F11)	0.960	0.945	0.953	0.962	0.930	0.947	0.863
(F32)	0.958	0.941	0.949	0.958	0.926	0.942	0.859
↑ Local NCC							
(F11)	0.740	0.741	0.771	0.773	0.660	0.753	0.642
(F32)	0.724	0.734	0.762	0.764	0.663	0.741	0.635
↓ LMSE							
(F11)	0.008	0.010	0.006	0.007	0.014	0.010	0.020
(F32)	0.008	0.011	0.008	0.007	0.014	0.011	0.020
↑ SI							
(F11)	0.846	0.681	0.850	0.852	0.800	0.836	0.760
(F32)	0.841	0.665	0.843	0.843	0.795	0.822	0.752
↓ BRISQUE							
(F11)	27.455	38.415	39.612	37.113	39.023	39.356	40.770
(F32)	27.335	37.671	38.407	36.312	37.747	37.425	38.682
↓ NIQE							
(F11)	3.840	3.881	4.200	3.826	4.297	4.164	4.246
(F32)	3.473	3.702	4.009	3.688	3.989	3.995	3.980
↓ PIQE							
(F11)	55.338	69.400	66.964	66.949	68.589	68.773	67.613
(F32)	54.818	65.918	61.951	62.797	65.273	64.330	63.523

Color coding: Green = best, Orange = second-best.

**Table 10:** Evaluation of proposed method compared to the non-learning methods using indoor data set with T-variance. ↑ and ↓ denote the higher and lower values are better, respectively.

Tvar	Proposed	LB14 [13]	WS16 [29]	NR17 [25]	YW19 [7]
↑ PSNR					
(T3)	21.485	12.074	15.235	15.549	16.242
(T10)	21.440	12.066	15.142	15.487	16.763
↓ MSE					
(T3)	0.008	0.081	0.064	0.062	0.060
(T10)	0.008	0.080	0.065	0.062	0.060
↑ SSIM					
(T3)	0.831	0.449	0.511	0.521	0.522
(T10)	0.834	0.447	0.512	0.522	0.523
↑ Global NCC					
(T3)	0.957	0.540	0.545	0.543	0.548
(T10)	0.957	0.539	0.543	0.542	0.546
↑ Local NCC					
(T3)	0.722	0.421	0.351	0.386	0.380
(T10)	0.723	0.417	0.351	0.386	0.380
↓ LMSE					
(T3)	0.008	0.080	0.068	0.067	0.067
(T10)	0.008	0.081	0.068	0.068	0.067
↑ SI					
(T3)	0.828	0.479	0.494	0.517	0.509
(T10)	0.831	0.479	0.497	0.518	0.510
↓ BRISQUE					
(T3)	27.416	26.371	41.008	39.156	36.840
(T10)	27.544	26.923	40.983	39.424	37.110
↓ NIQE					
(T3)	3.546	3.397	4.454	4.937	4.089
(T10)	3.522	3.468	4.416	4.968	4.133
↓ PIQE					
(T3)	54.443	35.409	56.112	55.588	50.629
(T10)	54.851	34.912	56.023	55.791	50.663

Color coding: Green = best, Orange = second-best.

**Table 11:** Evaluation of proposed method compared to the deep learning methods using indoor data set with T-variance. ↑ and ↓ denote the higher and lower values are better, respectively.

Tvar	Proposed	CoRRN [44]	ZN18 [36]	YD18 [37]	ERRNet [45]	SIRRBL [46]	IBCLN [47]
↑ PSNR							
(T3)	21.485	19.251	22.478	23.006	20.382	22.309	18.624
(T10)	21.440	19.001	22.372	23.029	20.426	22.174	18.551
↓ MSE							
(T3)	0.008	0.018	0.006	0.006	0.010	0.006	0.014
(T10)	0.008	0.018	0.006	0.006	0.010	0.007	0.015
↑ SSIM							
(T3)	0.831	0.705	0.840	0.856	0.793	0.825	0.715
(T10)	0.834	0.702	0.841	0.853	0.796	0.825	0.712
↑ Global NCC							
(T3)	0.957	0.945	0.949	0.957	0.928	0.944	0.864
(T10)	0.957	0.942	0.949	0.958	0.927	0.942	0.861
↑ Local NCC							
(T3)	0.722	0.735	0.762	0.768	0.661	0.742	0.641
(T10)	0.723	0.736	0.764	0.765	0.664	0.743	0.637
↓ LMSE							
(T3)	0.008	0.011	0.008	0.007	0.014	0.010	0.020
(T10)	0.008	0.012	0.008	0.007	0.014	0.011	0.020
↑ SI							
(T3)	0.828	0.664	0.842	0.848	0.794	0.823	0.754
(T10)	0.831	0.667	0.842	0.842	0.795	0.821	0.748
↓ BRISQUE							
(T3)	27.416	38.545	39.283	37.023	37.834	37.869	40.441
(T10)	27.544	38.420	39.353	37.404	38.570	38.056	39.213
↓ NIQE							
(T3)	3.546	3.864	4.091	3.726	4.156	4.130	4.173
(T10)	3.522	3.861	4.039	3.765	4.035	4.070	4.035
↓ PIQE							
(T3)	54.443	66.609	63.295	63.405	65.625	65.206	64.425
(T10)	54.851	66.391	62.714	62.522	65.205	64.849	64.216

Color coding: Green = best, Orange = second-best.

## Data Availability

The data presented in this study used the ‘SIR2+’ Dataset made available by the ROSE Lab at the Nanyang Technological University, Singapore. The dataset can be directly downloaded from the openly available repository: https://sir2data.github.io/ (accessed on 22 May 2026).
